# Water‐Enabled Ultralong Full‐Color Organic Phosphorescence in Hydrogen‐Bonded Frameworks for 4D Encryption and Bio‐Imaging

**DOI:** 10.1002/advs.76094

**Published:** 2026-06-19

**Authors:** Pengcheng Wu, Zenggang Lin, Lu Yang, Weisheng Liu

**Affiliations:** ^1^ The Second Hospital & Clinical Medical School Department of Ophthalmology Lanzhou University Lanzhou P. R. China; ^2^ Gansu Province Clinical Research Center For Ophthalmology Lanzhou P. R. China; ^3^ MOE Frontiers Science Center for Rare Isotopes Key Laboratory of Nonferrous Metal Chemistry and Resources Utilization of Gansu Province Engineering Research Center of Rare Earth Functional Materials Ministry of Education State Key Laboratory of Applied Organic Chemistry College of Chemistry and Chemical Engineering Lanzhou University Lanzhou P. R. China

**Keywords:** bioimaging, full‐color afterglow, host–guest assembly, hydrogen‐bonded organic frameworks, room temperature phosphorescence, water‐enhanced emission

## Abstract

Developing water‐stable organic room‐temperature phosphorescence (RTP) materials remains a formidable challenge due to water‐induced quenching. Herein, we present a counter‐intuitive strategy to achieve water‐enhanced ultralong RTP via the in situ encapsulation of carbonyl‐based guests within a rigid hydrogen‐bonded organic framework (HOF). Unlike conventional systems where water acts as a quencher, we demonstrate that water molecules function as pivotal structural reinforcers. Mechanistic studies reveal that water bridges host–guest hydrogen‐bonding sites, constructing a denser network that rigidifies molecular conformation and promotes intersystem crossing. Consequently, phosphorescence intensity peaks at a high water content of 55 wt.%. Significantly, this universal strategy enables full‐color ultralong phosphorescence (from blue to deep red) by tuning guest conjugation‐a rare feat in aqueous media. Benefiting from exceptional physiological stability and biocompatibility, these nano‐sized hybrids overcome intracellular quenching bottlenecks, enabling high‐signal‐to‐noise ratio cellular bio‐imaging. Additionally, applications in 4D encryption and humidity sensing are demonstrated. This work transforms a traditional phosphorescence killer into a synergistic enhancer, offering a novel paradigm for designing high‐performance RTP materials tailored for biological applications.

## Graphical abstract

Water‐induced quenching challenges organic RTP materials, but a counter‐intuitive strategy employs HOF to encapsulate carbonyl guests, where water functions as a structural reinforcer. This achieves water‐enhanced ultralong RTP with full‐color emission (blue to deep red), overcoming intracellular quenching for high‐signal cellular imaging, alongside 4D encryption and humidity sensing. A novel paradigm for bio‐applicable RTP materials.

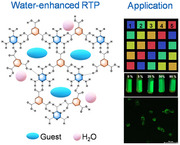

## 1 Introduction

Compared with inorganic long‐afterglow materials, which often suffer from poor biocompatibility and harsh preparation conditions, organic room‐temperature phosphorescence (RTP) materials have garnered significant attention due to their facile synthesis, mild processing conditions, and tunable optical properties [1, 2]. Over the past few decades, researchers have successfully realized long‐lived organic RTP by employing strategies such as crystal engineering, covalent polymerization, host–guest doping, and H‐aggregation [3, 4, 5, 6, 7]. These approaches generally promote intersystem crossing (ISC) by enhancing spin‐orbit coupling (SOC) and suppress non‐radiative transitions by constructing rigid microenvironments [8, 9]. Consequently, these unique RTP materials have demonstrated tremendous potential in fields such as information encryption, anti‐counterfeiting, and bio‐imaging [10, 11, 12, 13, 14]. However, developing RTP materials that possess water‐stimuli responsiveness or maintain stability in aqueous phases remains a formidable challenge [15]. Although there have been reports of achieving RTP in water using organic nanoparticles, macrocyclic supramolecules, or polymer matrices [16, 17, 18, 19], the realization of water‐enhanced RTP remains elusive [20]. The core difficulty lies in the fact that water molecules easily disrupt intermolecular hydrogen bonds, exacerbating molecular motion and leading to severe non‐radiative decay [21, 22]; simultaneously, dissolved oxygen readily quenches triplet excitons. Therefore, developing a universal strategy to construct water‐enhanced RTP materials is of significant importance for expanding their applications in advanced encryption and biological imaging.

It is well‐established that constructing a rigid environment can effectively restrict molecular motion, shield against quenchers, and improve ISC efficiency, while intermolecular interactions (such as hydrogen bonding and *π–π* stacking) help stabilize triplet excitons [23, 24, 25, 26]. Hydrogen‐bonded Organic Frameworks (HOFs), as an emerging class of crystalline porous materials, are comparable to MOFs and COFs in terms of structural diversity and architectural flexibility, while offering superior processability [27]. Based on these attributes, HOFs are expected to serve as ideal carriers for achieving water‐stable and enhanced RTP [28]. On one hand, strong hydrogen‐bonding interactions between the HOF host–guest molecules can boost the RTP performance of the chromophores. More critically, the abundant hydrogen‐bonding sites within the HOF skeleton can synergistically interact with water molecules to construct a novel hydrogen‐bonding network, thereby further enhancing the RTP performance of the system [29, 30].

**SCHEME 1:**
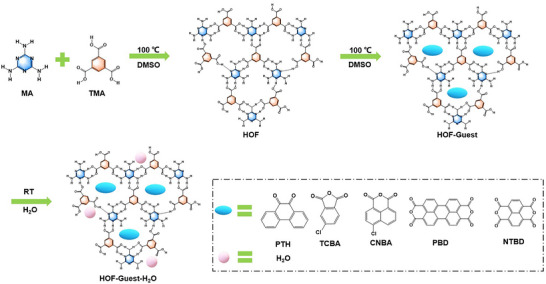
Schematic illustration of the in situ synthesis process for HOF and HOF‐Guest doped materials.

In this study, we employed an efficient and universal in situ synthesis strategy to successfully prepare water‐enhanced RTP materials with phosphorescence lifetimes reaching hundreds of milliseconds (Scheme 1). By in situ self‐assembling melamine (MA) and 1,3,5‐benzenetricarboxylic acid (TMA) to form a porous HOF skeleton, and co‐assembling a series of carbonyl‐containing organic phosphors, we not only achieved precise control over the material size but also utilized the rigid environment provided by the HOF to significantly suppress the non‐radiative decay of the guest molecules. Notably, by modulating the conjugation degree of the guest, we induced an emission redshift, realizing full‐color, naked‐eye visible phosphorescence ranging from blue to deep red. More uniquely, the introduction of an appropriate amount of water (55 wt.%) did not cause quenching; instead, it significantly enhanced the RTP intensity by constructing an additional hydrogen‐bonding network and facilitating efficient ISC channels. Benefiting from the stability of the HOF skeleton in pure water as well as strongly acidic and alkaline environments, this series of materials exhibits low toxicity and excellent dispersibility in biological imaging (OCM‑1 cells), and demonstrates broad application prospects in 4D encryption and reversible humidity sensing.

## 2 Results and Discussion

### 2.1 Preparation and Structural Characterization of HOF‐Guest Materials

To establish a versatile and robust strategy for water‐enhanced RTP materials, we first conducted a detailed characterization of the morphology and structure. As shown in Figure S1, scanning electron microscopy (SEM) images reveal that the pristine HOF monomers exhibit typical rod‐like microcrystalline structures, with a tendency to aggregate into micron‐sized particles. Upon encapsulating a series of guest molecules into the HOF matrix, the morphology of the resulting doped materials remained largely consistent with the pristine HOF, demonstrating that the doping process did not significantly disrupt the microscopic morphology. Regarding the crystal structure, as depicted in Figure S2a, the powder X‐ray diffraction (PXRD) patterns of the series of HOF‐Guest doped materials align well with that of the pristine HOF, confirming the high crystallinity of the framework. The variations in peak intensities can be attributed to changes in preferred orientation induced by guest encapsulation rather than framework collapse. Furthermore, thermogravimetric analysis (TGA) indicates that all HOF‐Guest doped materials maintain excellent thermal stability up to 300°C (Figure S2b), further corroborating that the HOF matrix retains its structural integrity after guest encapsulation. Furthermore, to unambiguously confirm the porous nature of the as‐prepared HOF, nitrogen adsorption–desorption isotherms were measured at 77 K. As depicted in Figure S3, the HOF exhibits a Type I isotherm, confirming its microporous structure, with a calculated BET specific surface area of 19.2570 m^2^/g. For the HOF‐PTH, HOF‐TCBA, HOF‐CNBA, HOF‐PBD, and HOF‐NTBD that adsorb guest molecules, their BET specific surface areas are 6.6273, 14.6574, 10.0647, 9.2655, and 17.7834 m^2^/g, respectively, all lower than that of HOF. This significant reduction indicates that guest molecules successfully occupy the pore channels of HOF, reducing the effective surface area available for nitrogen adsorption, which further validates the porosity of the HOF framework and the interaction between guest molecules and the framework.

Fourier transform infrared spectroscopy (FT‐IR) provided molecular‐level evidence for the successful preparation of the doped materials (Figure S4). Strong hydrogen‐bonding interactions between the aromatic carbonyl groups of the guest molecules and the HOF matrix significantly restrict the stretching motions of the host's ─NH_2_ groups, leading to a noticeable redshift in their stretching vibration frequencies: the characteristic peaks originally located at 3523 and 3364 cm^−1^ in the HOF matrix shifted to approximately 3396 and 3294 cm^−1^, respectively, after encapsulating the series of guest molecules. Additionally, X‐ray photoelectron spectroscopy (XPS) further elucidated the host–guest interaction mechanism (Figure S5). By deconvoluting the N1s and O1s spectra of HOF, the O1s spectrum exhibits three characteristic peaks assigned to C═O (531.49 eV), O─H (532.45 eV), and C─O (533.55 eV) bonds; the N1s spectrum can be resolved into two binding energy peaks corresponding to C═N─C (399.24 eV) on the triazine ring and amino N─H (400.40 eV). Comparing the fitting results of the series of HOF‐Guest doped materials, it is evident that the binding energies of the relevant chemical bonds shifted to varying degrees after successful guest encapsulation. These shifts in XPS binding energies strongly confirm the formation of an enhanced non‐covalent hydrogen‐bonding network between the guest molecules and the HOF matrix, rather than the generation of new covalent bonds. This is consistent with the FT‐IR results, confirming the successful encapsulation of guest molecules within the HOF channels and the integrity of the matrix structure. Collectively, the above characterization results comprehensively verify the successful encapsulation of guest molecules in HOF, as well as the structural integrity and stability of the HOF matrix before and after encapsulation.

### 2.2 Photophysical Properties of HOF‐Guest Doped Materials

The doping concentration of guest molecules is a critical factor governing the RTP performance of host–guest materials. To determine the optimal preparation conditions, we systematically investigated the influence of varying guest doping molar ratios (0.5%, 1.0%, 2.0%, 3.0%, 5%, and 10%) on the emission properties, using HOF‐CNBA as a representative model. As illustrated in Figure S6, the phosphorescence intensity exhibited a significant enhancement trend as the CNBA doping ratio gradually increased from 0.5% to 2.0%. However, with a further increase in the doping ratio, the phosphorescence intensity began to decline, which is primarily attributed to the concentration quenching effect caused by the aggregation of guest molecules at high concentrations. Concurrently, the variation trend of phosphorescence lifetime was highly consistent with the emission intensity: the lifetime first increased and then decreased with the doping concentration (Figure S6b), indicating that excessive guest molecules lead to aggregation‐induced concentration quenching and an increase in non‐radiative decay channels. Based on these experimental results, 2.0% was determined as the optimal doping molar ratio, and a series of HOF‐Guest doped materials (HOF‐PTH, HOF‐TCBA, HOF‐CNBA, HOF‐PBD, and HOF‐NTBD) were prepared accordingly for subsequent studies.

**FIGURE 1:**
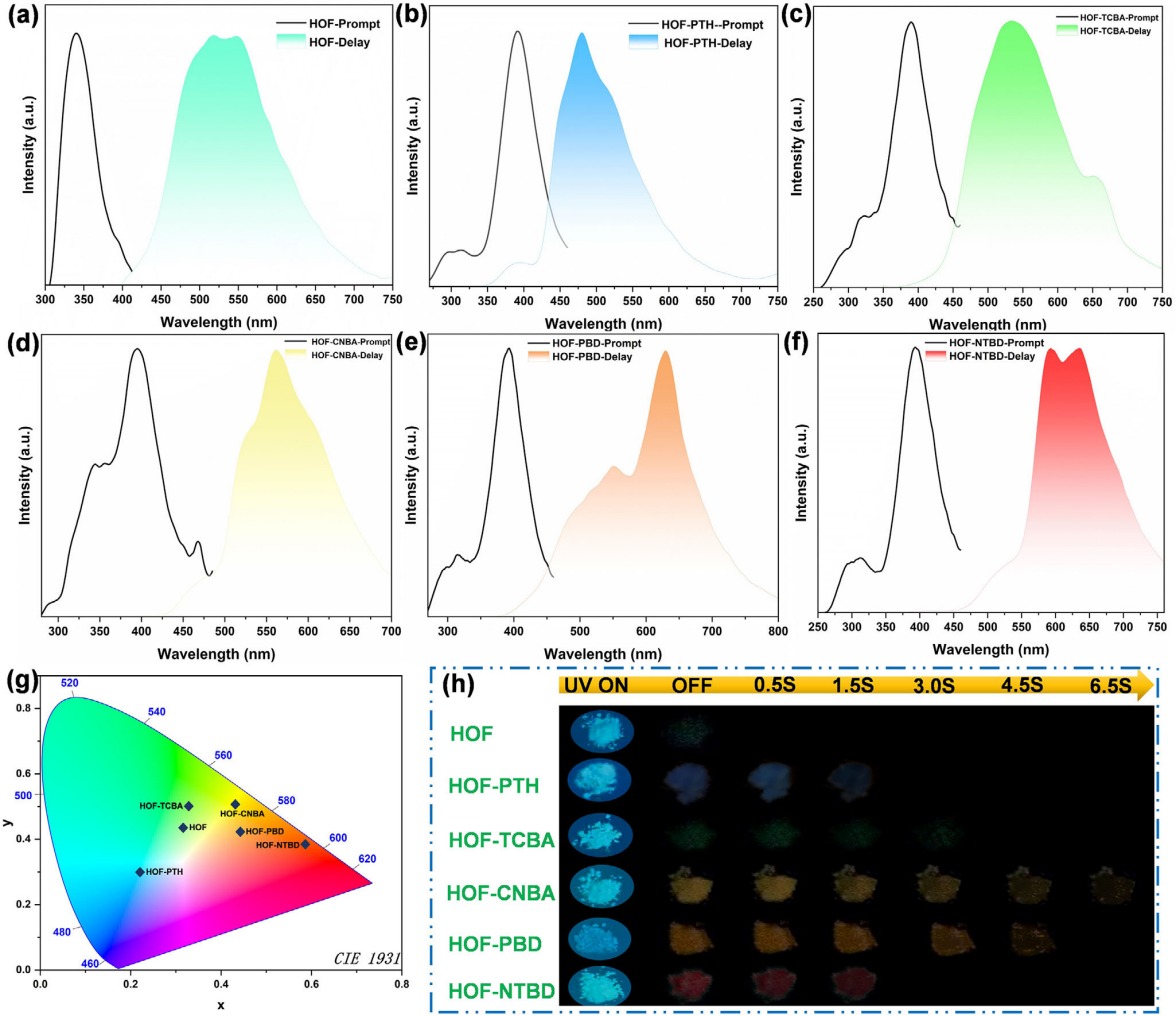
(a–f) Normalized steady‐state photoluminescence (Prompt) and delayed phosphorescence (Delay) spectra of HOF, HOF‐PTH, HOF‐TCBA, HOF‐CNBA, HOF‐PBD, and HOF‐NTBD at room temperature. (g) CIE 1931 chromaticity diagram showing the phosphorescence coordinates of the HOF host and HOF‐guest doped materials. (h) Time‐resolved photographs of the corresponding powders before and after the cessation of UV irradiation. Excitation wavelengths: 340 nm for HOF, HOF‐PTH, and HOF‐TCBA; 254 nm for the others.

To evaluate the regulatory role of the HOF encapsulation strategy on optical properties, we compared the emission characteristics of the series of doped materials with those of pristine HOF. As shown in Figure 1a, pristine HOF exhibits a fluorescence emission peak at 340 nm and dual phosphorescence emission peaks at 516 and 548 nm. Its average RTP lifetime at 548 nm is 42.73 ms (Figure S7c), presenting a naked‐eye visible cyan afterglow (Figure 1h). In contrast, after guest encapsulation, the fluorescence emission peaks of the doped materials redshifted by approximately 55 nm, accompanied by the emergence of a shoulder peak around 330 nm, likely due to potential energy transfer and energy level matching between the HOF host–guest molecules [31]. Regarding phosphorescence emission, the host–guest system demonstrated exceptional tunability. HOF‐PTH emitted bright blue phosphorescence at 480 nm, exhibiting a visible afterglow of approximately 2 s to the naked eye (Figure 1b). HOF‐TCBA showed green phosphorescence at 520 nm (Figure 1c); HOF‐CNBA displayed yellow phosphorescence at 560 nm (Figure 1d); HOF‐PBD presented orange phosphorescence with dual emission peaks at 550 and 630 nm (Figure 1e); and HOF‐NTBD exhibited deep‐red phosphorescence at 590 and 630 nm (Figure 1f). As the conjugation system of the guest molecules gradually extended from PTH to NTBD, the phosphorescence emission peaks of the doped materials underwent significant redshifts, successfully achieving full‐color phosphorescence emission spanning from blue to deep red (confirmed by CIE coordinates in Figure 1g). It is particularly noteworthy that achieving deep‐red RTP with emission wavelengths exceeding 700 nm is highly challenging, yet the materials in this study exhibited this rare property [32]. Furthermore, as shown in Figure S7 and Table S1, benefiting from the in situ encapsulation strategy, both the phosphorescence lifetimes and quantum yields of the doped materials were significantly enhanced compared to pristine HOF. This proves that this strategy not only effectively broadens the emission gamut but also markedly boosts the luminous efficiency of the materials.

To unravel the physical mechanism underlying the ultralong RTP emission, we compared the luminescence behavior of guest molecules in different environments. As depicted in Figure S8, the guest molecules exhibited only negligible phosphorescence at room temperature, whereas their phosphorescence intensity was dramatically enhanced at a low temperature of 77 K. The high overlap between the room‐temperature emission peaks of the HOF‐Guest materials and the intrinsic phosphorescence peaks of the guests at 77 K provides critical evidence that the HOF matrix offers a rigid confined environment akin to cryogenic conditions for the guest molecules. This effectively freezes molecular vibrations and rotations, thereby activating room‐temperature phosphorescence emission.

Further excitation spectral analysis elucidated the origin of the luminescence centers. As shown in Figure S9, although the transient excitation spectra appear similar, the delayed excitation spectra reveal significant differences: with the introduction of guests, the dominant excitation peak gradually shifted from 340 nm (characteristic of the HOF host) to 260–270 nm (characteristic absorption of the guests). This confirms that the RTP emission mainly originates from guest molecules tightly immobilized by the rigid HOF matrix. Moreover, wavelength‐dependent measurements (Figure S10) showed that as the excitation wavelength redshifted from 254 to 340 nm, the intensity of materials dominated by HOF matrix emission (e.g., HOF‐PTH) increased, while the intensity of materials dominated by guest emission (e.g., HOF‐NTBD) significantly decreased. Collectively, the RTP emission primarily stems from guest molecules rigidly fixed within the HOF matrix. The in situ encapsulation strategy realizes high‐quantum‐yield ultralong room‐temperature phosphorescence emission by suppressing non‐radiative transitions and stabilizing triplet excitons.

### 2.3 Water‐Enhanced Photophysical Properties

**FIGURE 2:**
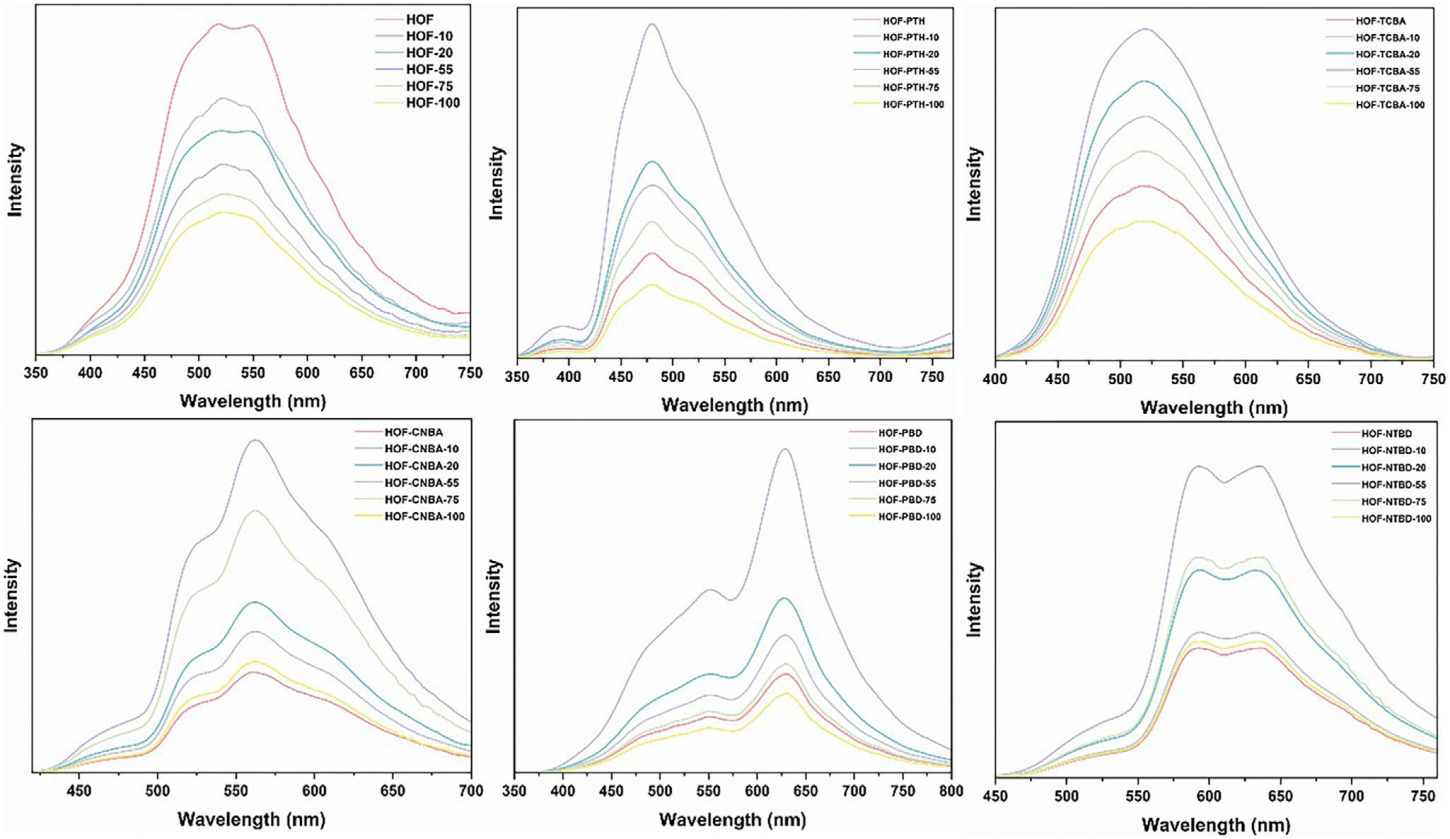
(a–f) Delayed phosphorescence spectra of HOF, HOF‐PTH, HOF‐TCBA, HOF‐CNBA, HOF‐PBD, and HOF‐NTBD with varying water contents (0–100 wt.%) at room temperature.

It is well‐known that dissolved oxygen and water molecules typically disrupt intermolecular hydrogen bonds and accelerate the non‐radiative decay of triplet excitons, leading to the quenching of most organic RTP materials in aqueous phases. However, this study reveals that the abundant potential hydrogen‐bonding sites within the HOF skeleton can generate a strong synergistic effect with water molecules, thereby inducing anomalous phosphorescence enhancement at low water contents. As shown in Figure 2 and Figure S11, the phosphorescence of pristine HOF was rapidly quenched with increasing water content (almost disappearing at 20 wt.%). In stark contrast, the guest‐encapsulated doped materials exhibited a significant phosphorescence enhancement upon the introduction of water, reaching a peak intensity at 55 wt.% water content, and maintaining strong emission even at a high water content of 70 wt.%.

To quantitatively characterize the water‐enhanced RTP phenomenon, we employed a modified Stern‐Volmer equation to fit the luminescence enhancement curves of the guest‐encapsulated HOF materials. As shown in Figure S12, the relationship between the relative phosphorescence intensity (I/I_0_) and the mass ratio of water to material ([*C*]) was accurately described by the equation (I/I_0_ = 1 + K*a*[*C*]) [33], exhibiting highly linear correlations (R^2^ >0.99) for all materials within the linear range of 0%–55% water content. This high linearity demonstrates the feasibility of these materials for quantitative humidity detection.

The robustness and reproducibility of this water‐enhanced RTP effect were further confirmed through cyclic phosphorescence intensity measurements under 55 wt.% water conditions after vacuum drying (Figure S13). For pristine HOF, the phosphorescence intensity experienced a sharp decrease after water addition and exhibited continuous decay over 5 cycles, confirming the water‐induced quenching effect. In contrast, all guest‐encapsulated HOF materials (HOF‐PTH, HOF‐TCBA, HOF‐CNBA, HOF‐PBD, and HOF‐NTBD) displayed notable phosphorescence enhancement in the presence of water, with the intensity remaining stable over 5 cycles. This indicates that water functions as a synergistic structural reinforcer instead of a quencher, and the water‐enhanced RTP effect is robust and reproducible. This enhancement is not attributed to a change in the luminescent center (the emission wavelength remains largely unchanged) but rather arises from the rigidification of the microenvironment induced by water molecules.

Further photophysical characterizations elucidated the underlying mechanism (Figure S14 and Table S2): the quantum yield of HOF‐CNBA increased consistently with the emission intensity as the water content rose (showing a dramatic boost at 55 wt.%), while its phosphorescence lifetime remained essentially constant. This critical result indicates that water molecules do not merely fill the pores; more importantly, they construct a new hydrogen‐bonding network that facilitates the intersystem crossing (ISC) process of the guest molecules. This significantly enhances the radiative transition probability, confirming the central role of guest molecules in the water‐enhanced RTP phenomenon.

Based on the superior properties described above, we further investigated the stability of the materials in fully aqueous environments and under extreme pH conditions. As shown in Figure S15, although the phosphorescence intensity decreased slightly once the water content exceeded the optimal value, the series of doped materials maintained stable, full‐color phosphorescence emission even in fully aqueous suspensions. Even more encouragingly, these materials demonstrated exceptional acid and alkali resistance: in HCl solution (pH = 2) or NaOH solution (pH = 12), the emission peak positions remained unchanged with only a negligible decrease in intensity. Furthermore, long‐term monitoring of the dispersions revealed no apparent decay over a period of 7 days (Figure S15g). This indicates that the rigid HOF matrix provides a robust protective shield for the guest molecules, effectively blocking quenchers in harsh aqueous environments without the need for additional deoxygenation, thereby preserving structural integrity and excellent optical performance.

To elucidate the microscopic mechanism underlying the water‐enhanced RTP, we conducted a comprehensive analysis combining structural characterization with theoretical calculations. XRD and DSC results reveal that the introduction of water induced subtle expansion and adjustment of the material structure (Figure S16). The appearance of water‐related endothermic peaks in the low‐temperature regions (4°C and 45°C–100°C) confirms the formation of strong hydrogen‐bonding interactions between water molecules and the HOF skeleton [34]. Here, water molecules act as “bridges” connecting the HOF framework and the guest molecules, significantly increasing the system's rigidity and suppressing non‐radiative transitions.

**FIGURE 3:**
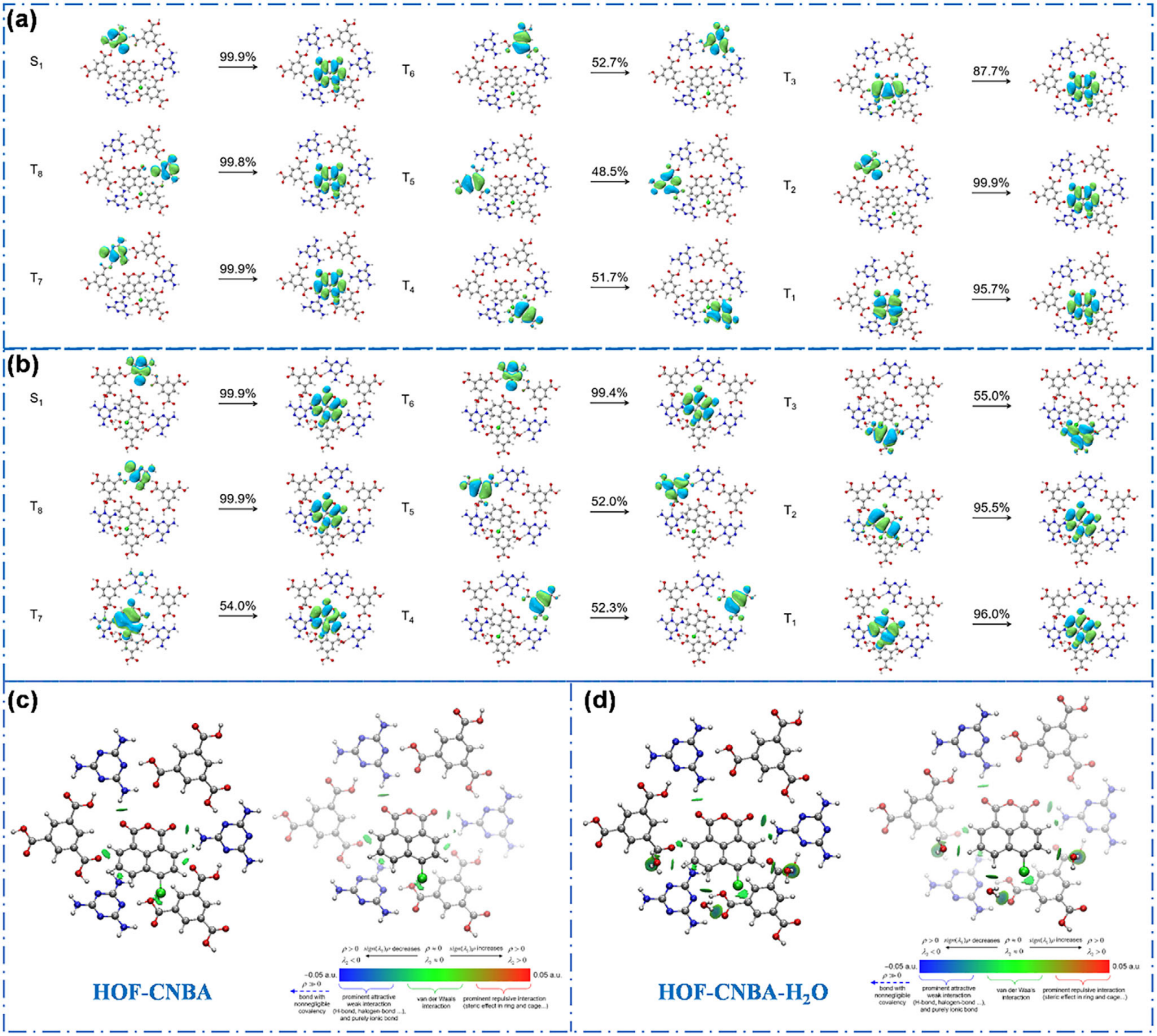
Theoretical insights into the water‐enhanced RTP mechanism. (a,b) Natural transition orbitals (NTOs) representing the hole (left) and electron (right) distributions for selected excited states involved in the intersystem crossing (ISC) channels for (a) HOF‐CNBA and (b) HOF‐CNBA‐H_2_O. The percentage values indicate the contribution weights of the dominant orbital pairs. (c,d) Reduced density gradient (RDG) analyses of non‐covalent interactions in (c) HOF‐CNBA and (d) HOF‐CNBA‐H_2_O cluster models.

Theoretical calculations further elucidated the electronic‐level mechanism of this enhancement. As shown in Figure 3a,b, and Figure S17, TD‐DFT calculations indicate that although the introduction of water did not significantly alter the spin‐orbit coupling (SOC) constants (explaining the stability of the lifetime), it effectively promoted the intersystem crossing (ISC) process by narrowing the singlet‐triplet energy gap (Δ*E_ST_*) and increasing the overlap integral of natural transition orbitals (NTOs). Consistent with these theoretical predictions, low‐temperature (77 K) fluorescence and phosphorescence spectroscopy measurements further confirmed the reduction of Δ*E_ST_* upon hydration [35]. As shown in Figure S18, HOF‐CNBA exhibits fluorescence and phosphorescence peaks at 393 and 529 nm, respectively, corresponding to a Δ*E_ST_* of 0.82 eV. In contrast, HOF‐CNBA‐55 shows fluorescence and phosphorescence peaks at 415 and 467 nm, respectively, resulting in a significantly reduced Δ*E_ST_* of 0.33 eV. This notable decrease in Δ*E_ST_* upon hydration directly supports our proposed mechanism: water‐mediated hydrogen‐bonding networks effectively narrow the energy gap between the singlet and triplet states, thereby facilitating ISC and enhancing RTP efficiency.

Furthermore, visualization analysis based on the Independent Gradient Model based on Hirshfeld partitioning (IGMH) intuitively demonstrated that the intervention of water molecules resulted in more extensive and stronger non‐covalent interaction regions between the HOF and the guest (Figure 3c,d) [36]. In summary, the one‐pot in situ encapsulation strategy not only firmly locks the guest molecules within the rigid HOF cavities but also ingeniously leverages water molecules to construct a dense hydrogen‐bonding network. This synergistically suppresses molecular motion and activates efficient ISC channels, thereby realizing the counter‐intuitive water‐enhanced ultralong phosphorescence.

### 2.4 Multifunctional Applications: From 4D Anti‐Counterfeiting Encryption and Humidity Sensing to Bio‐Imaging

**FIGURE 4:**
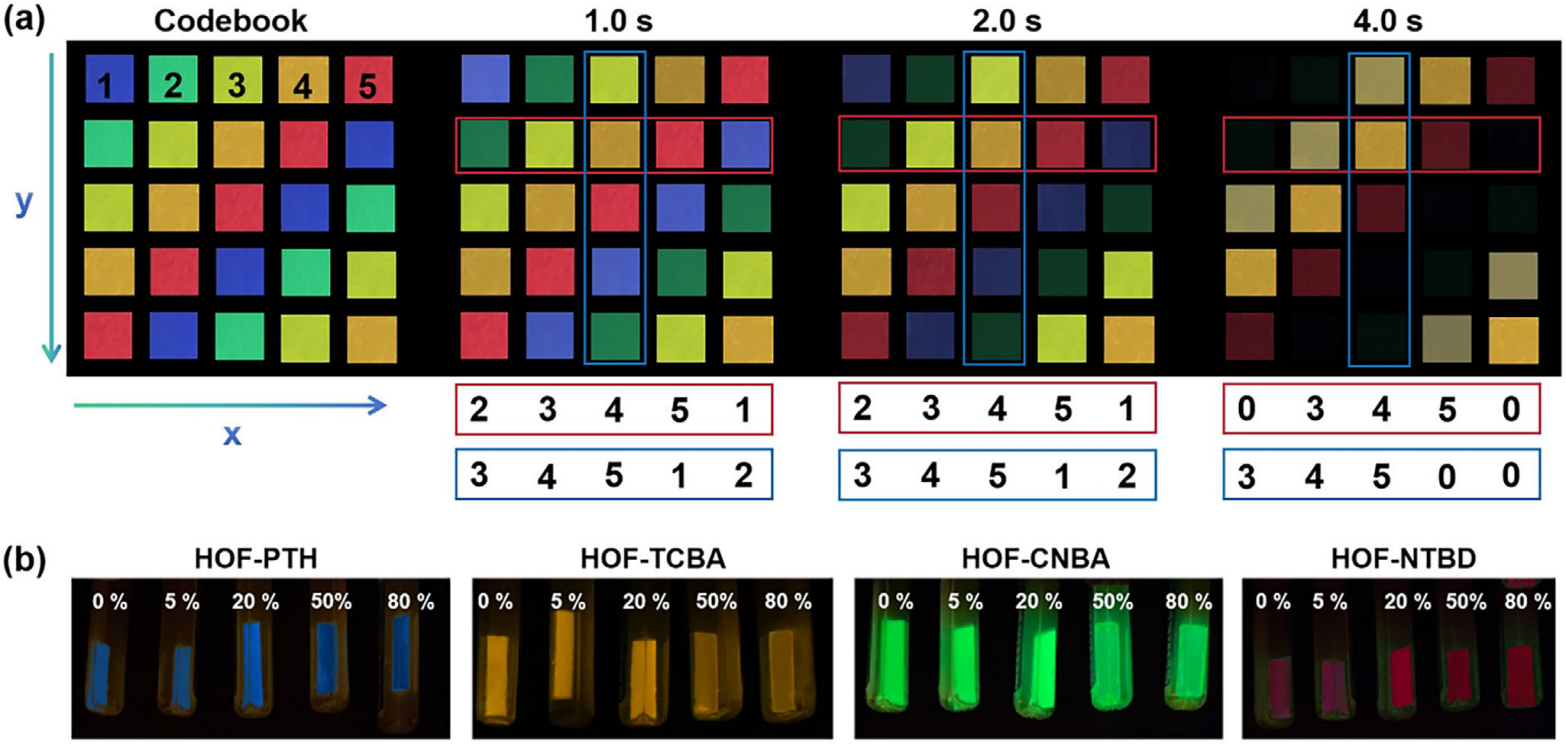
(a) Schematic illustration of the 4D encryption and decryption process based on time‐resolved phosphorescence colors. The decoded numbers in the *x*‐direction (red box) and *y*‐direction (blue box) evolve dynamically over time (1.0, 2.0, and 4.0 s) due to the distinct phosphorescence lifetimes of different guest molecules. (b) Photographs of humidity test strips coated with HOF‐PTH, HOF‐TCBA, HOF‐CNBA, and HOF‐NTBD under varying relative humidity (RH) levels (0%–80%) after the cessation of UV irradiation.

Benefiting from the full‐color phosphorescence emission characteristics and superior stability in aqueous environments of the HOF‐Guest doped materials, we have expanded their application scope to multifunctional fields, ranging from advanced anti‐counterfeiting to biomedical imaging. First, to overcome the limitations of low information capacity and poor security inherent in traditional 2D “on‐off” encryption modes, we introduced the dimension of “time” and constructed a high‐order 4D encryption model by combining the rich emission colors (spectral dimension) with the differentiated lifetime characteristics of the materials (Figure 4a). By defining distinct emission colors as numerical codes (Blue = 1, Green = 2, Yellow = 3, Orange = 4, Red = 5, and Black = 0), the information read from the *x*‐ and *y*‐axis decryption zones during the short‐lived stage (1.0–2.0 s after cessation of UV excitation) corresponds to “23451” and “34512”, respectively. However, in the long‐lived stage after 4.0 s, as the short‐lived blue and green components extinguish first and transition to “0”, the decoded information dynamically flips to “03450” and “34500”. This spatiotemporally resolved dynamic information display significantly enhances anti‐counterfeiting security.

Second, leveraging the remarkable phosphorescence enhancement effect at low water content, we developed high‐sensitivity humidity detection test strips (Figure 4b). Benefiting from the large specific surface area provided by the filter paper fiber structure, which facilitates effective contact between trace water molecules and the active sites of the material, the test strips can induce the formation of a rigid hydrogen‐bonding network and reach naked‐eye visible saturation brightness at a humidity level as low as 20%. More importantly, given the stable luminescence properties of these materials in aqueous physiological environments, we further explored their potential in the field of bio‐imaging.

Furthermore, given the robust luminescence stability of these materials in aqueous physiological environments, we delved into their application prospects in the field of biomedical imaging. First, to meet the stringent size requirements for biological applications, we successfully downsized the HOF‐doped materials to the nanoscale by finely tuning the reaction kinetics via a low‐temperature anti‐solvent precipitation strategy, as detailed in the Experimental Section. Transmission electron microscopy (TEM) comparisons before and after regulating the reaction conditions clearly evidence this size reduction (Figures S19 and S20). Dynamic light scattering (DLS) measurements further confirmed the successful downsizing, revealing an average hydrodynamic diameter in the nanoscale range with a moderate polydispersity index (PDI) of approximately 0.3 (Figure S21). Notably, the DLS profile remained largely unchanged after 5 days of dispersion, verifying the excellent colloidal stability of the nano‐sized HOF‐Guest materials in physiological environments (Figure S22). This nanoscale dimension and robust stability significantly enhanced their cellular uptake efficiency and biocompatibility.

**FIGURE 5:**
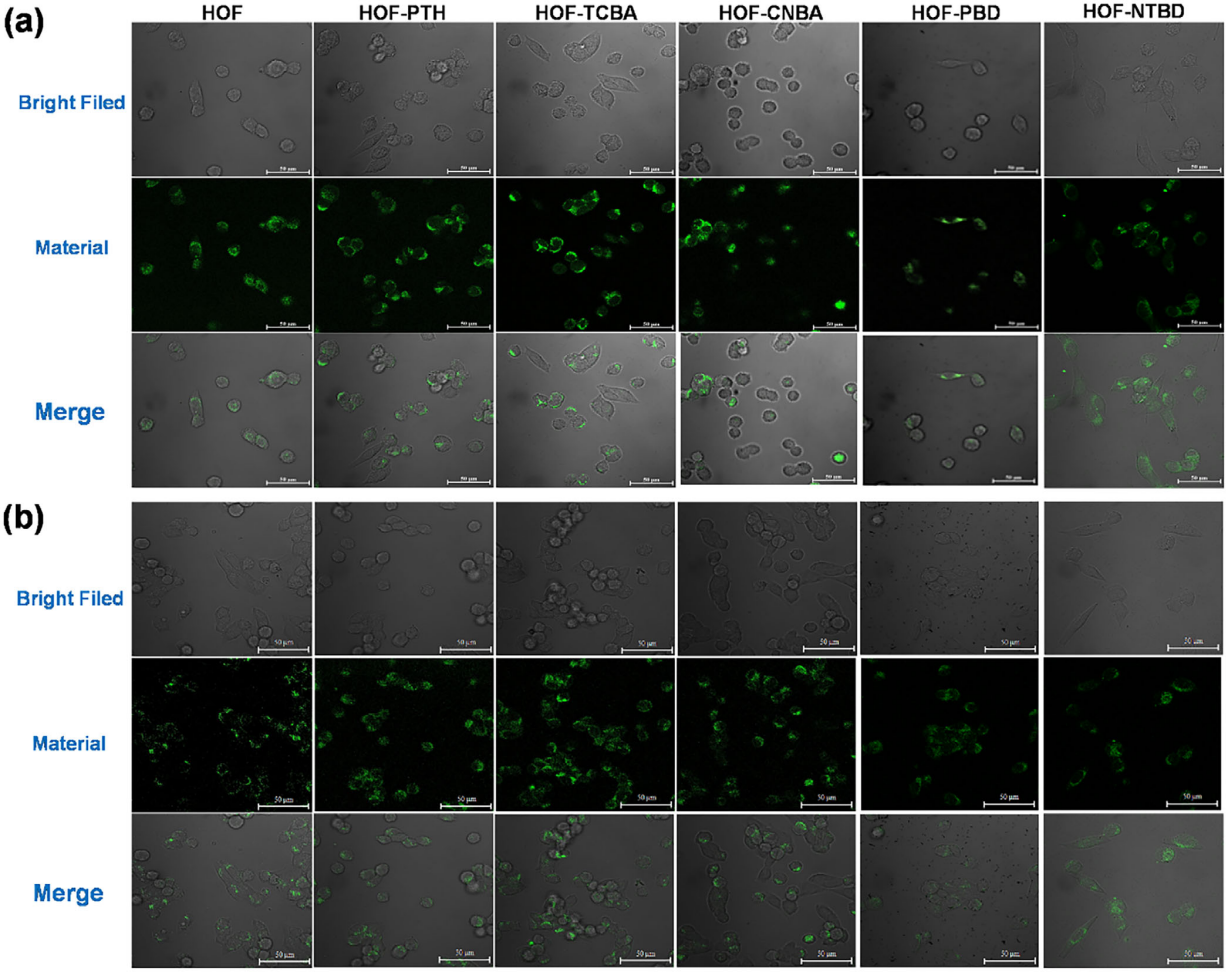
Confocal laser scanning microscopy (CLSM) images of OCM‐1 human choroidal melanoma cells after co‐incubation with HOF and the series of HOF‐Guest doped materials. (a) Fluorescence imaging mode. Top row: Bright‐field images; Middle row: Fluorescence images; Bottom row: Merged images of bright‐field and fluorescence. (b) Phosphorescence imaging mode (acquired with a 1 ms delay). Top row: Bright‐field images; Middle row: Phosphorescence images; Bottom row: Merged images of bright‐field and phosphorescence.

Subsequently, human choroidal melanoma cells (OCM‐1) were selected as a model to evaluate the biosafety of the materials. Cytotoxicity tests revealed that OCM‐1 cells maintained a high viability rate even after co‐incubation with the materials at a high concentration of 1000 µg/mL, confirming the extremely low cytotoxicity and excellent biocompatibility of the materials (Figure S23). Confocal laser scanning microscopy (CLSM) imaging results (Figure 5a) clearly demonstrated that cells incubated with the materials maintained a healthy spindle‐shaped adherent morphology and intact membrane structures under bright‐field observation, further corroborating the low cytotoxicity of these materials. Concurrently, confocal imaging revealed that green fluorescence signals were homogeneously distributed throughout the cytoplasm without significant aggregation. This observation confirms that the nanosized HOF‐Guest materials can efficiently enter cells via endocytosis, thereby enabling bioimaging with a high signal‐to‐noise ratio and minimal background interference. Furthermore, phosphorescence confocal imaging with a delay of 1 ms (Figure 5b) clearly demonstrated the phosphorescence imaging capability of this system in physiological environments. The phosphorescence signals exhibited long‐lived emission characteristics and effectively resisted autofluorescence interference, overcoming the inherent limitations of conventional fluorescence imaging. This dual‐mode imaging approach, combining both fluorescence and phosphorescence, offers enhanced imaging contrast and shows great potential for deep‐tissue imaging. In conclusion, this series of materials not only provides novel perspectives for information encryption and environmental sensing but also holds broad application prospects in the field of bio‐theranostics, particularly in scenarios requiring high‐contrast and long‐term tracking.

## 3 Conclusion

In summary, we have developed a versatile in situ host–guest assembly strategy to construct a series of water‐enhanced organic RTP materials using a HOF matrix. Breaking conventional wisdom, we demonstrated that water molecules can act as synergistic structural reinforcers rather than quenchers, significantly boosting phosphorescence efficiency by constructing a denser hydrogen‐bonding network. This unique mechanism not only enabled the realization of tunable full‐color ultralong afterglow but also endowed the materials with exceptional stability in physiological environments. Consequently, we successfully demonstrated their practical utility in advanced 4D information encryption and, notably, high‐contrast cellular bio‐imaging with low background interference. This work provides a paradigm shift for designing robust RTP systems, bridging the gap between fundamental theories and real‐world aqueous applications.

## Author Contributions

**Pengcheng Wu**: conceptualization, funding acquisition, methodology, software, writing – review and editing, formal analysis, data curation. **Weisheng Liu**: conceptualization, funding acquisition, writing – review and editing, validation, investigation, supervision, data curation. **Zenggang Lin**: conceptualization, methodology, investigation, writing – original draft, writing – review and editing, visualization, software, data curation. **Lu Yang**: funding acquisition, methodology, writing – review and editing, supervision, validation.

## Conflicts of Interest

The authors declare no conflicts of interest.

## Supporting information




**Supporting File**: advs76094‐sup‐0001‐SuppMat.docx.

## Data Availability

The data that support the findings of this study are available in the supplementary material of this article.
