# Supplementary material for: Water‐Enabled Ultralong Full‐Color Organic Phosphorescence in Hydrogen‐Bonded Frameworks for 4D Encryption and Bio‐Imaging
Source: Adv Sci (Weinh). 2026 Jun 19:e76094. Online ahead of print. doi: 10.1002/advs.76094 (PMC13336675; doi:10.1002/advs.76094)
Supplement: Supplementary file 1 — Supporting File: advs76094‐sup‐0001‐SuppMat.docx. [file ADVS-9999-e76094-s001.docx]

Supplementary information

**Water-Enabled Ultralong Full-Color Organic Phosphorescence in Hydrogen-Bonded Frameworks for 4D Encryption and Bio-imaging**

*Pengcheng Wu,^[a,†]^ Zenggang Lin,^[a,b,†]1^ Lu Yang,^*[a]^ and Weisheng Liu^*[b]^*

[a] P. Wu, Z. Lin, L. Yang

The Second Hospital & Clinical Medical School, Department of Ophthalmology, Gansu Province Clinical Research Center for Ophthalmology,

Lanzhou University

Lanzhou, 730000 (P. R. China)

[b] Z. Lin, W. Liu

MOE Frontiers Science Center for Rare Isotopes, Key Laboratory of Nonferrous Metal Chemistry and Resources Utilization of Gansu Province, Engineering Research Center of Rare Earth Functional Materials, Ministry of Education, State Key Laboratory of Applied Organic Chemistry, College of Chemistry and Chemical Engineering

Lanzhou University

Lanzhou, 730000 (P. R. China)

E-mail: [luyang21@lzu.edu.cn](mailto:luyang21@lzu.edu.cn)(L. Yang), [liuws@lzu.edu.cn](mailto:liuws@lzu.edu.cn)(W. Liu)

**†Pengcheng Wu, and Zenggang Lin contributed equally to this work.**

**1. Experimental Section**

**1.1 Experimental Instruments**

Fourier transform infrared (FT-IR) spectra were recorded on a Thermo Scientific Nicolet iS5 spectrometer. The Brunauer-Emmett-Teller (BET) specific surface area was determined using a Micromeritics ASAP 2460 instrument. X-ray photoelectron spectroscopy (XPS) was performed using a Kratos AXIS SUPRA+ spectrometer. Differential scanning calorimetry (DSC) and thermogravimetric analysis (TGA) were conducted on a TGA/DSC3+/Sartorius/MCA125S-2CCN-1 instrument. XRD patterns were recorded on a Rigaku D/Max‑2400 X‑ray diffractometer. Dynamic light scattering (DLS) measurements were performed on a Malvern Zetasizer Nano ZS nanoparticle size and Zeta potential analyzer. Photoluminescence (PL) spectra and lifetimes were recorded on an FLS980 fluorescence spectrometer (Edinburgh Instruments Ltd.) equipped with a xenon arc lamp (Xe900), a microsecond flash lamp (μF900), and an Oxford OptistatDN for temperature control. The photoluminescence quantum yields (φ) of solid‑state samples were measured on an FLS1000 fluorescence spectrometer (Edinburgh Instruments Ltd.) with an integrating sphere. Photographs of the luminescence were taken using a mobile phone. Confocal laser scanning microscopy images were captured using a Nikon Ti2-E confocal laser scanning microscope.The absorbance in the cell compatibility assay was measured with an Agilent BioTek Synergy H1 multi-mode microplate reader.

**1.2 Materials**

Melamine and phenanthrenequinone were purchased from Macklin Biochemical Technology Co., Ltd.1,3,5-Benzenetricarboxylic acid, 4-chlorophthalic anhydride, 4-chloro-1,8-naphthalic anhydride, perylene-3,4,9,10-tetracarboxylic dianhydride, and 1,4,5,8-naphthalenetetracarboxylic dianhydride were purchased from Saan Chemical Technology (Shanghai) Co., Ltd. All solvents were purchased from Tianjin Hengxing Chemical Reagent Manufacturing Co., Ltd., China. The CCK-8 kit was purchased from APExBIO, USA.

**1.3 Preparation and Synthesis of Materials**

**1.3.1 Synthesis of HOF**

Equimolar amounts of melamine (MA, 0.5 g, 3.96 mmol) and 1,3,5-benzenetricarboxylic acid (TMA, 0.83 g, 3.96 mmol) were separately dissolved in 10 mL of DMSO to form clear solutions. The two solutions were then combined and stirred at 100 °C for 1 h, during which MA and TMA self-assembled via hydrogen-bonding interactions. After cooling to room temperature, the resulting solution was slowly poured into 50 mL of deionized water. The mixture was stirred continuously for 3 h to afford a white suspension. The precipitate was collected by centrifugation, washed thoroughly with deionized water to remove residual DMSO, and dried under vacuum to yield the HOF material as a white powder. Yield: 72%.

**1.3.2 Preparation of HOF-Guest Doped Materials**

The HOF-Guest doped materials were synthesized following a procedure similar to that of the pristine HOF, except that the respective guest molecules were introduced during the self-assembly process. The employed guest molecules included phenanthrenequinone (PTH), 4-chlorophthalic anhydride (TCBA), 4-chloro-1,8-naphthalic anhydride (CNBA), perylene-3,4,9,10-tetracarboxylic dianhydride (PBD), and 1,4,5,8-naphthalenetetracarboxylic dianhydride (NTBD). Yields at the optimal doping ratio: HOF-PTH (48%), HOF-TCBA (61%), HOF-CNBA (78%), HOF-PBD (58%), and HOF-NTBD (69%).

**1.3.3 Preparation of Small-Sized HOF and HOF-Guest Doped Materials**

Nano-sized HOF and HOF-Guest materials were synthesized via a low-temperature anti-solvent precipitation strategy. Typically, MA and TMA were separately dissolved in DMSO to afford 0.2 M stock solutions. For the HOF-Guest materials, the respective guest molecules were sonicated in ethanol to form uniform suspended stock solutions. Under ice-bath conditions, the DMSO stock solutions of MA and TMA alongside the guest suspended stock solution were simultaneously added dropwise into vigorously stirred ethanol (anti-solvent) at a rate of 0.5 mL/min. Subsequently, the mixture was heated to 60 °C and stirred for 30 min to facilitate framework crystallization. After the reaction, the resulting mixture was slowly poured into deionized water. The precipitates were collected by centrifugation, washed thoroughly, and dried to obtain uniform nanomaterials. Yields at the optimal doping ratio: HOF (54%), HOF-PTH (36%), HOF-TCBA (43%), HOF-CNBA (53%), HOF-PBD (44%), and HOF-NTBD (52%).

**1.3.4 Preparation of HOF-Guest-H_2_O Doped Materials**

To precisely control the water content within the HOF-Guest doped materials, a humidity-controlled equilibrium method was employed. A series of HOF-Guest samples were placed in desiccators containing saturated salt solutions, which provided specific relative humidity (RH) levels at a constant temperature. After equilibrating for 48 h, the water uptake of the samples was determined by the weighing method, yielding HOF-Guest-H_2_O with specific water contents. Specifically, HOF-Guest-25 represents a HOF-Guest doped material with an equilibrium water weight content of 25%. To ensure the water content remained constant during the measurements, all luminescence measurements were carried out in a sealed sample cell.

**1.4 Preparation of 4D Anti-counterfeiting Labels**

HOF-Guest doped material (10 mg) was dissolved in DMSO (1 mL) under heating. The solution was then uniformly mixed with water-based ink and drop-cast into a mold. The mixture was dried at 100 °C for 2 h to completely remove the DMSO solvent.

**1.5 Preparation of Humidity Test Paper**

HOF-Guest doped material (10 mg) was dissolved in DMSO (1 mL) under heating. The solution was uniformly coated onto filter paper and dried at 100 °C for 2 h to completely remove the DMSO solvent.

**1.6 Humidity Detection Process**

Different volumes of water were added to sealed plastic tubes according to specific volume ratios. The tubes were heated to convert the water into vapor, thereby establishing defined humidity environments for detection.

**1.7 Density Functional Theory (DFT) Calculations**

Density Functional Theory (DFT) calculations were performed using the Gaussian 16 program (revision D.01) [1]. The geometries of the materials at the lowest triplet excited state (T_1_) were optimized using the unrestricted DFT (UDFT) method at the B3LYP/6-31G(d) level. Based on the optimized T_1_ structures, time-dependent density functional theory (TDDFT) calculations were carried out at the M06-2X-D3/def2-SVP level to obtain the vertical excitation energies. The singlet and triplet excitation energies, along with the spin-orbit coupling (SOC) matrix elements, were calculated using the PySOC code [2]. Furthermore, TD-DFT calculations and Natural Transition Orbital (NTO) analyses were conducted at the B3LYP-D3(BJ)/def2-SVP level [3]. The Independent Gradient Model (IGM) analysis based on Hirshfeld partition was performed using the Multiwfn 3.8 (dev) code. The isosurface maps were visualized using Visual Molecular Dynamics (VMD 1.9.3) software based on the files exported from Multiwfn.

**1.8 Cell Culture**

Human ocular choroidal melanoma-1 (OCM-1) cells were retrieved from liquid nitrogen storage and rapidly thawed in a 37 °C water bath. The cell suspension was transferred to a 15 mL centrifuge tube and centrifuged at 1450 rpm for 10 min. After discarding the supernatant, 4 mL of complete cell culture medium containing 10% fetal bovine serum (FBS) and 1% penicillin-streptomycin was added, and the mixture was centrifuged again at 1450 rpm for 10 min. The supernatant was discarded, and the cells were resuspended in 1 mL of complete medium. The resuspended cells were seeded into a 25 cm^2^ cell culture flask and incubated in a humidified incubator at 37 °C with 5% CO2. After 2-3 days, the cells were observed under a microscope, and subculturing was performed when the cell confluence reached 80%-90%.

**1.9 In Vitro Biocompatibility**

The in vitro biocompatibility of HOFs and their doped materials was evaluated using the CCK-8 assay. To investigate the viability of OCM-1 tumor cells after co-incubation with the materials, 1 × 10^5^ cells were seeded into a 96-well plate containing 100 μL of complete medium and incubated at 37 °C with 5% CO_2_ for 24 h. Subsequently, 10 μL of material suspensions at different concentrations (0, 500, and 1000 μg/mL) were added to the wells, followed by incubation for another 24 h under the same conditions. The culture medium was then aspirated and discarded. After washing with PBS, 100 μL of fresh complete medium was added. Then, 10 μL of CCK-8 solution was added to each well, and the plate was returned to the incubator for 4 h. The absorbance of the samples at 450 nm was measured using a microplate reader.

**2. Supplementary Figures**


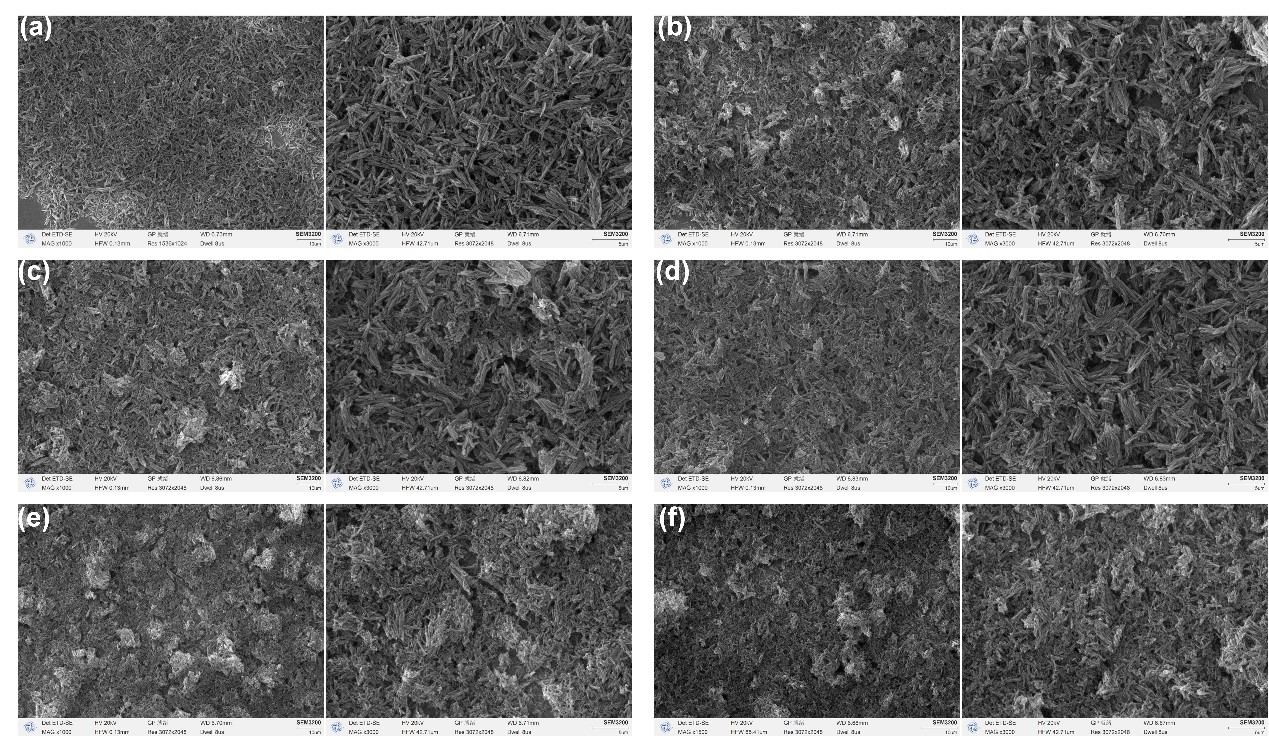


**Figure S1.** SEM images of (a) HOF, (b) HOF-PTH, (c) HOF-TCBA, (d) HOF-CNBA, (e) HOF-PBD, and (f) HOF-NTBD.


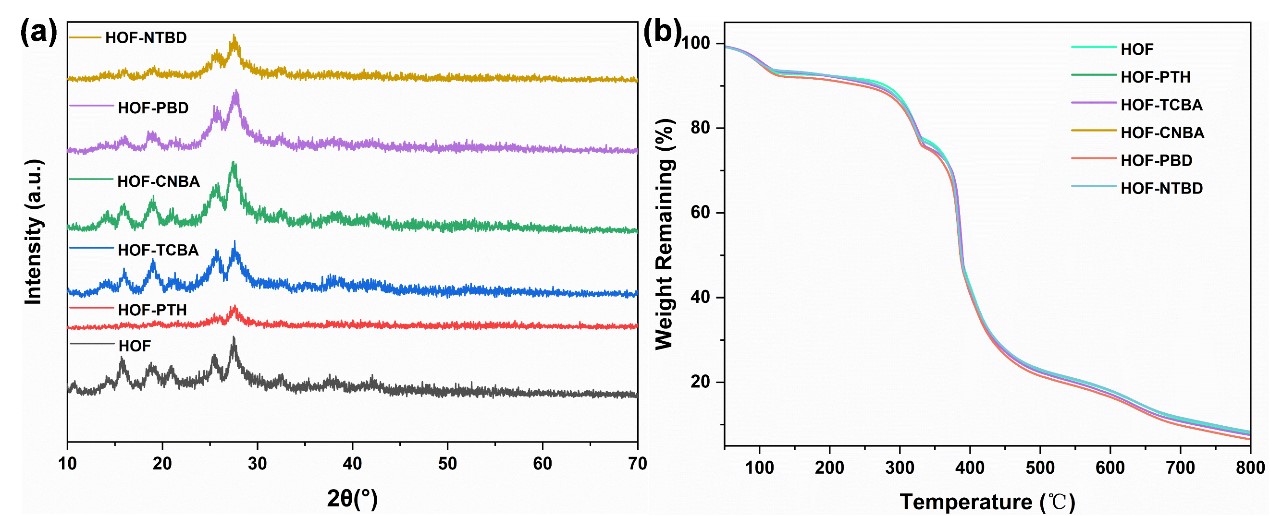


**Figure S2.** (a) XRD patterns of HOF and HOF-Guest doped materials; (b) TGA curves of HOF and HOF-Guest doped materials.

**
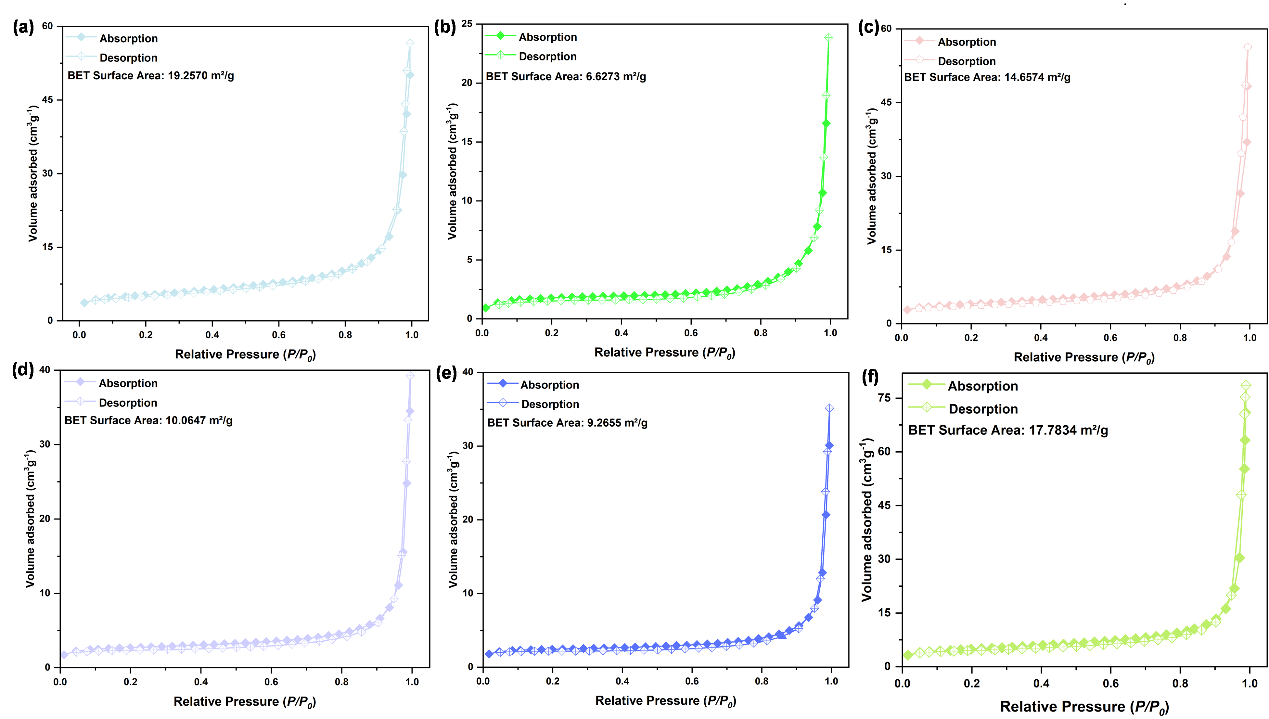
**

**Figure S3.** Nitrogen adsorption-desorption isotherms of (a) HOF, (b) HOF-PTH, (c) HOF-TCBA, (d) HOF-CNBA, (e) HOF-PBD, and (f) HOF-NTBD measured at 77 K. The BET specific surface areas are (a) 19.2570 m^2^/g, (b) 6.6273 m^2^/g, (c) 14.6574 m^2^/g, (d) 10.0647 m^2^/g, (e) 9.2655 m^2^/g, and (f) 17.7834 m^2^/g, respectively.


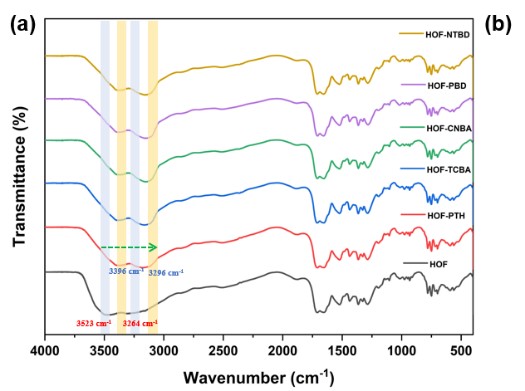


**Figure S4.** FT-IR spectra of HOF and HOF-Guest doped materials.


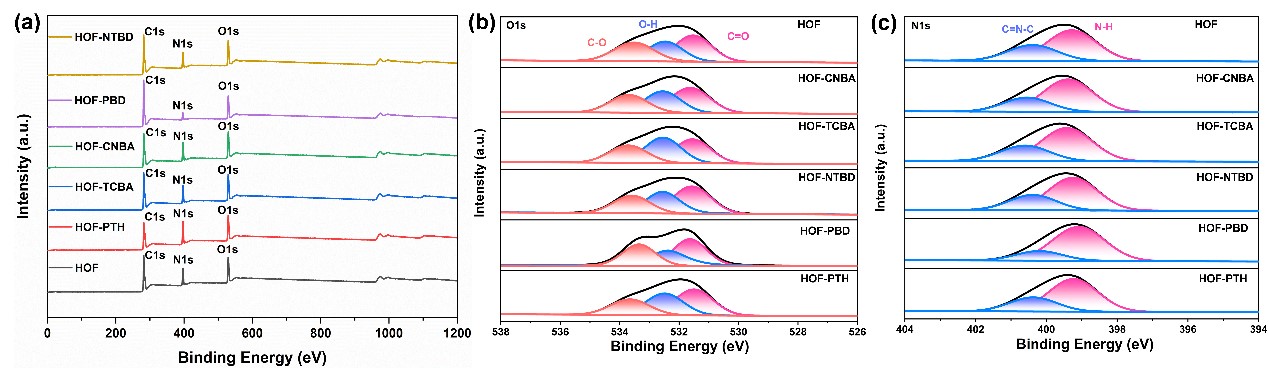


**Figure S5.** (a) XPS survey scans of HOF and HOF-Guest doped materials; (b) High-resolution O 1s XPS spectra of HOF and HOF-Guest doped materials; (c) High-resolution N 1s XPS spectra of HOF and HOF-Guest doped materials.


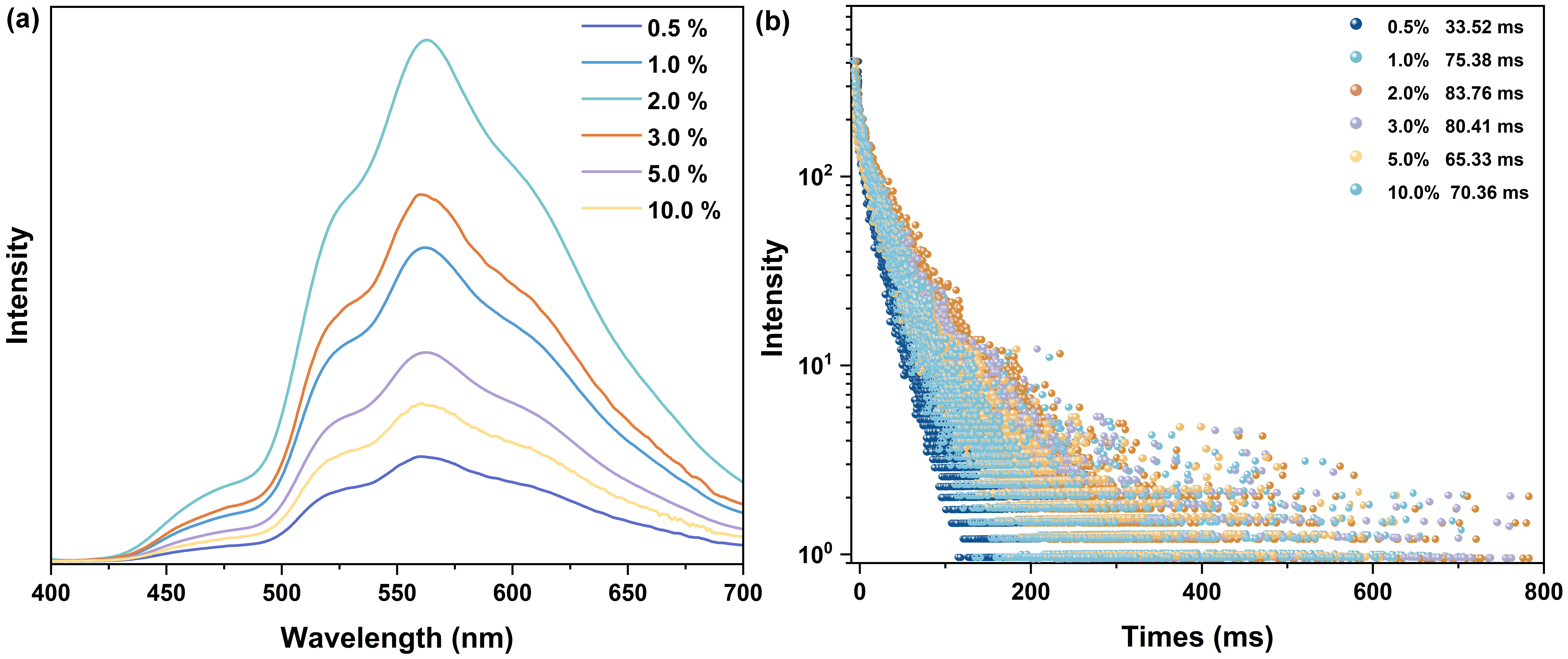


**Figure S6.** (a) Phosphorescence emission spectra (λex = 254 nm, delay = 8 ms) of HOF-CNBA materials containing different amounts of CNBA (molar ratio); (b) Phosphorescence decay curves of HOF-CNBA materials containing different amounts of CNBA (molar ratio).


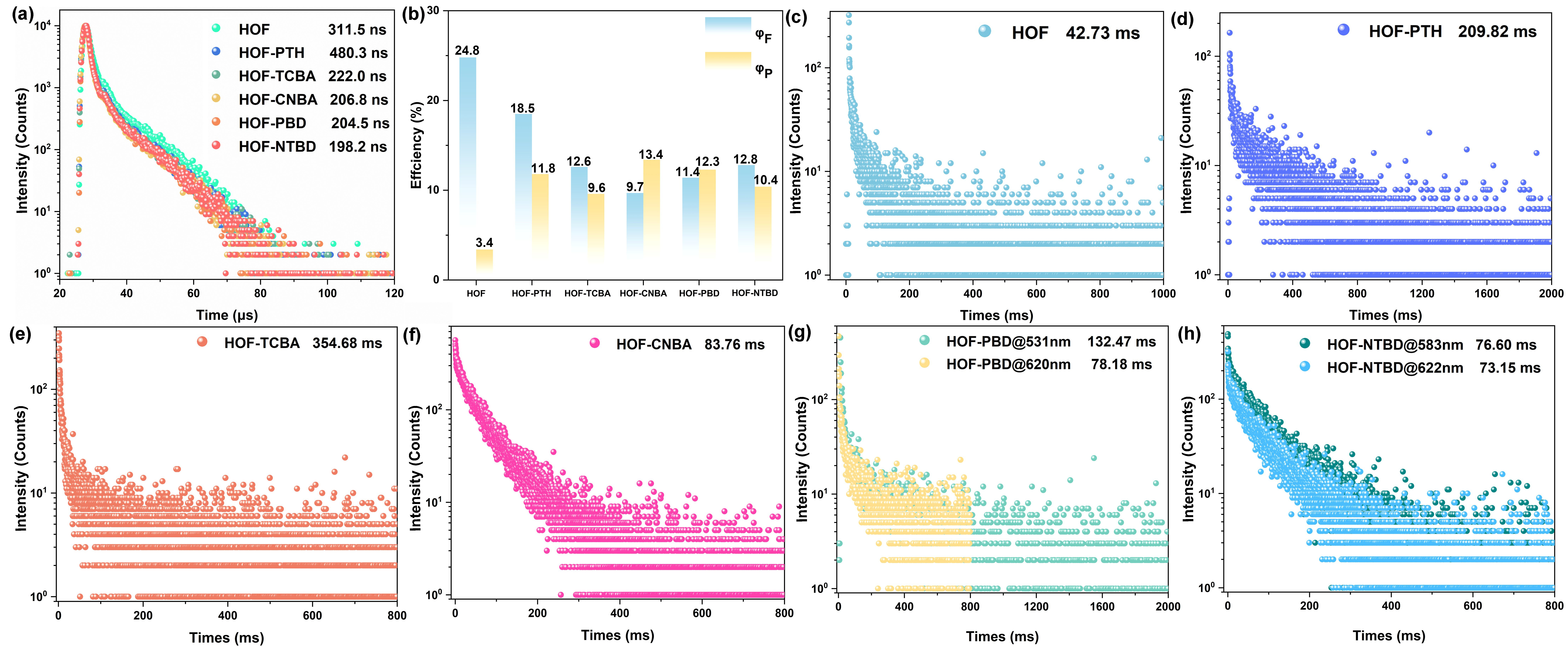


**Figure S7.** (a) Time-resolved fluorescence decay curves of HOF and HOF-Guest doped materials; (b) Fluorescence and phosphorescence quantum yields of HOF and HOF-Guest doped materials; Time-resolved phosphorescence decay curves of (c) HOF, (d) HOF-PTH, (e) HOF-TCBA, (f) HOF-CNBA, (g) HOF-PBD, and (h) HOF-NTBD.



**Figure S8.** (a) Fluorescence emission spectra and phosphorescence emission spectra of (b) HOF-PTH, (c) HOF-TCBA, (d) HOF-CNBA, (e) HOF-PBD, and (f) HOF-NTBD at room temperature and 77 K of the series of guest molecules.


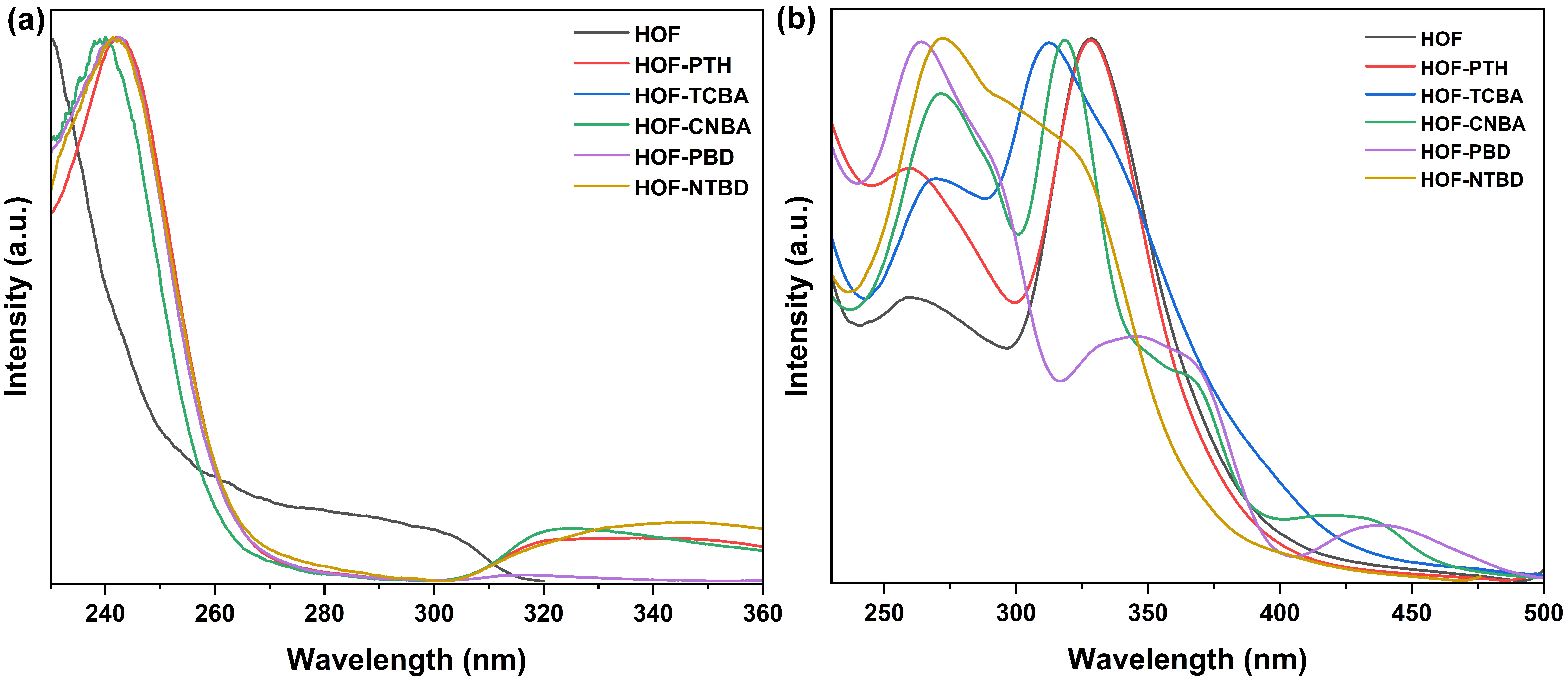


**Figure S9.** (a) Transient excitation spectra of HOF-Guest doped materials; (b) Delayed excitation spectra of HOF-Guest doped materials.





**Figure S10.** Phosphorescence emission spectra of (a) HOF, (b) PTH, (c) TCBA, (d) CNBA, (e) PBD, and (f) NTBD under different excitation wavelengths.


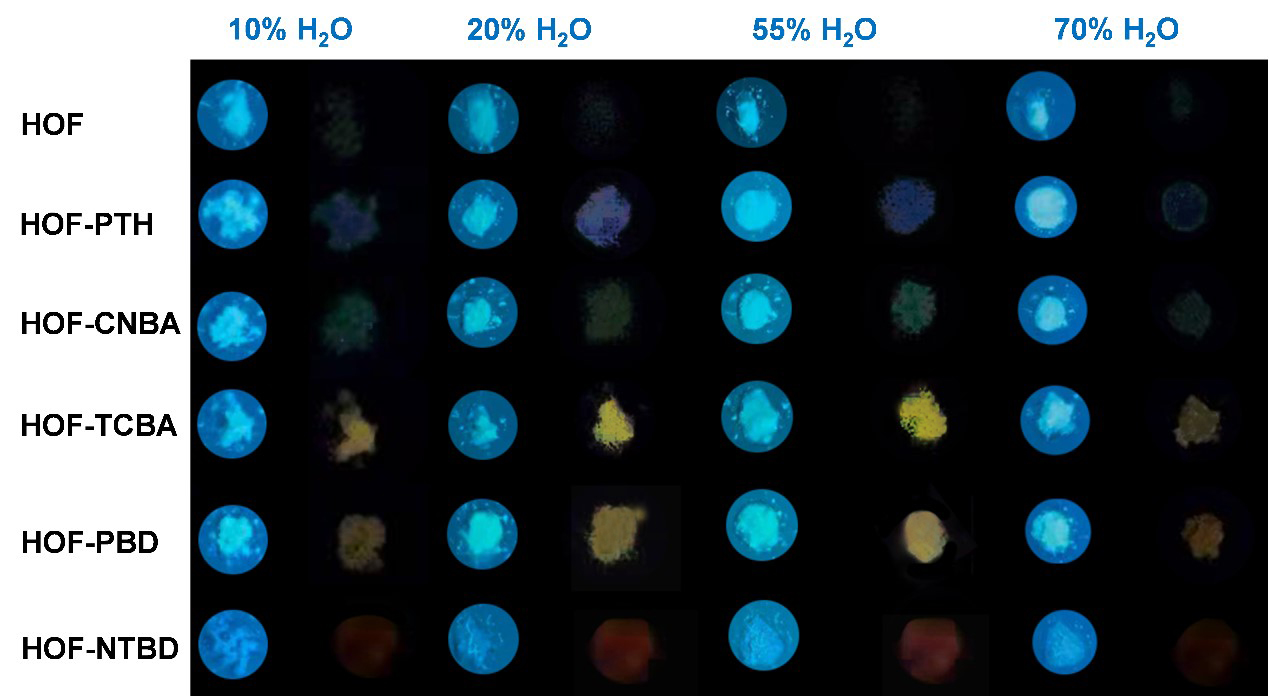


**Figure S11.** Photographs of HOF and HOF-Guest doped materials with different water contents under UV irradiation and at different time intervals after turning off the UV lamp.


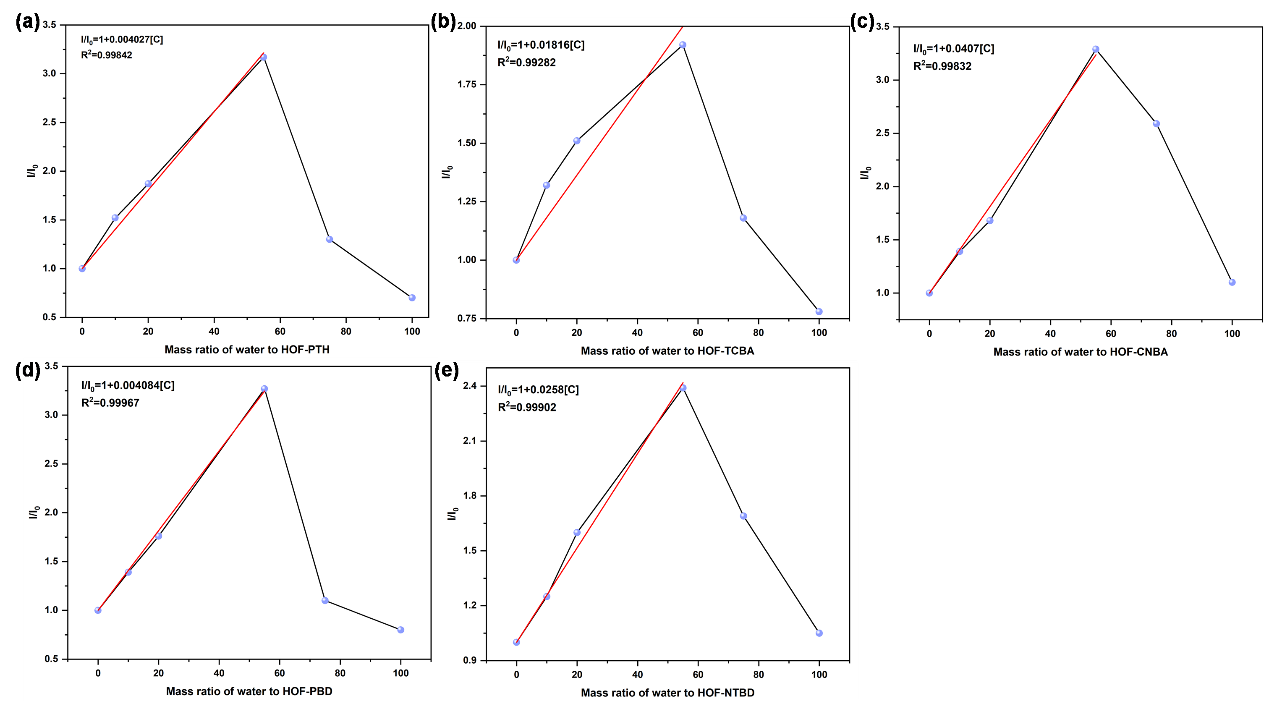


**Figure S12.** Linear fitting of luminescence enhancement for guest-encapsulated HOF materials (HOF-PTH, HOF-TCBA, HOF-CNBA, HOF-PBD, and HOF-NTBD) using a modified Stern-Volmer equation (I/I_0_ = 1 + K*a*[*C*]). The relationship between relative phosphorescence intensity (I/I_0_) and mass ratio of water to material ([*C*]) exhibits high linearity (R^2^ > 0.99) within the linear range of 0-55% water content, demonstrating the feasibility of these materials for quantitative humidity detection.


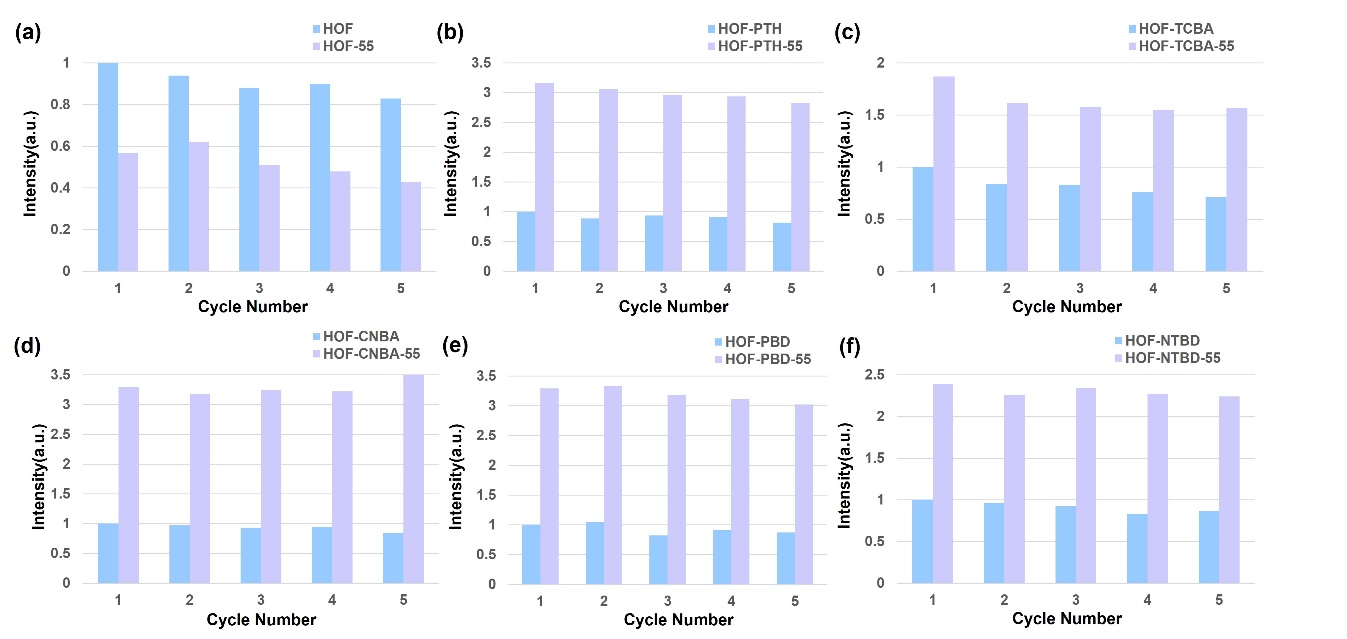


**Figure S13.** Cyclic phosphorescence intensity measurements of (a) HOF, (b) HOF-PTH, (c) HOF-TCBA, (d) HOF-CNBA, (e) HOF-PBD and (f) HOF-NTBD under dry and 55 wt.% water conditions. The samples were vacuum-dried before each cycle, and the intensity was recorded to evaluate the reversibility and robustness of the water-enhanced RTP effect.


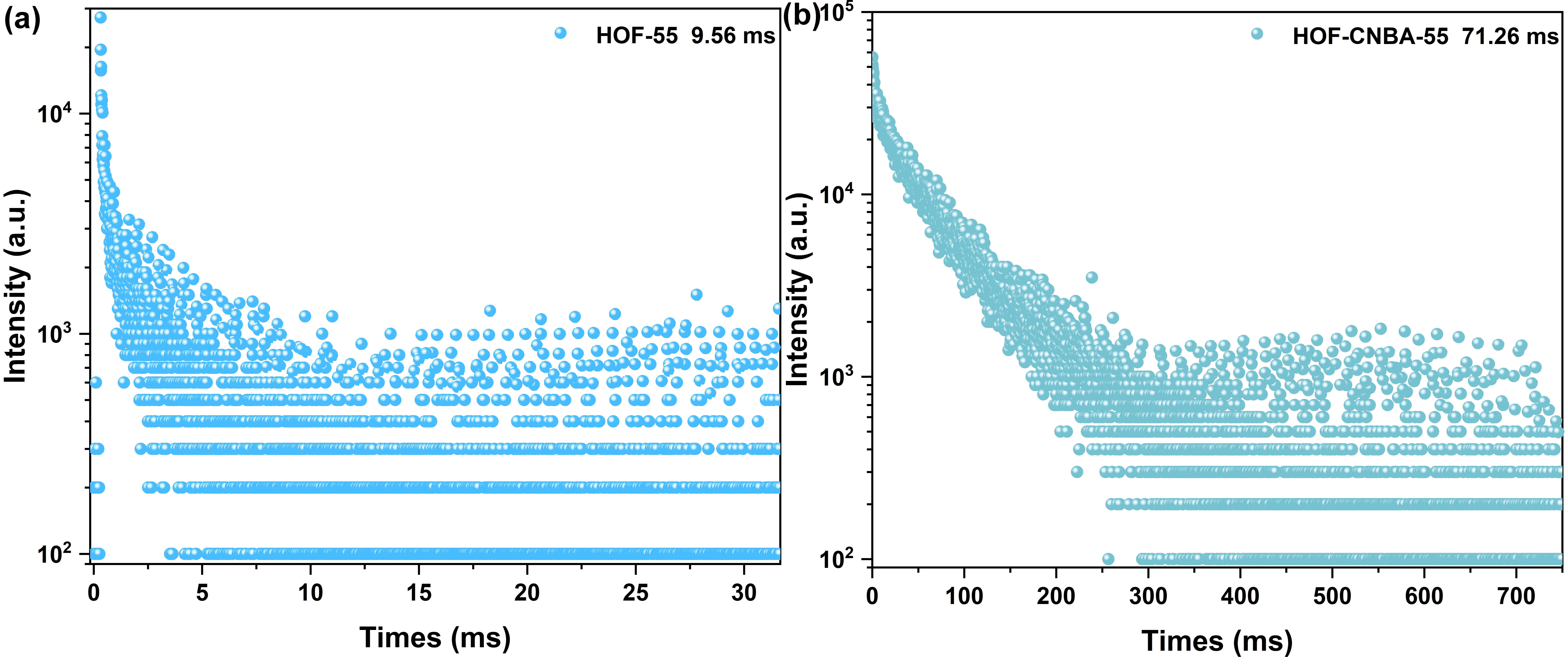


**Figure S14.** Phosphorescence decay curves of (a) HOF and (b) HOF-CNBA with a water content of 55%.


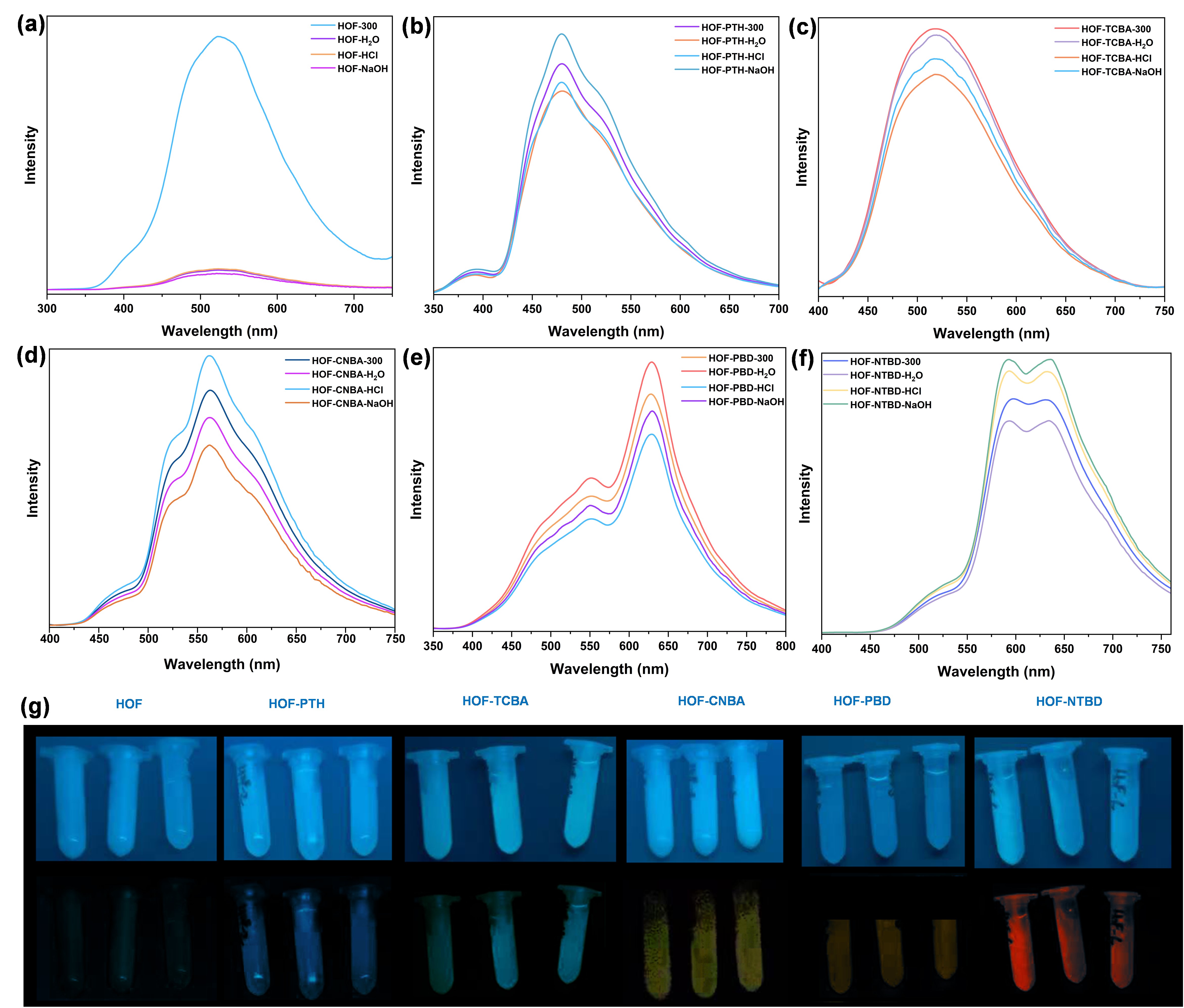


**Figure S15.** Phosphorescence emission spectra of (a) HOF, (b) HOF-PTH, (c) HOF-TCBA, (d) HOF-CNBA, (e) HOF-PBD, and (f) HOF-NTBD with different water contents and dispersed in strong acid/alkali solutions; (g) Photographs of HOF and HOF-Guest doped materials with different water contents and dispersed in strong acid/alkali solutions under UV irradiation and at different time intervals after turning off the UV lamp.


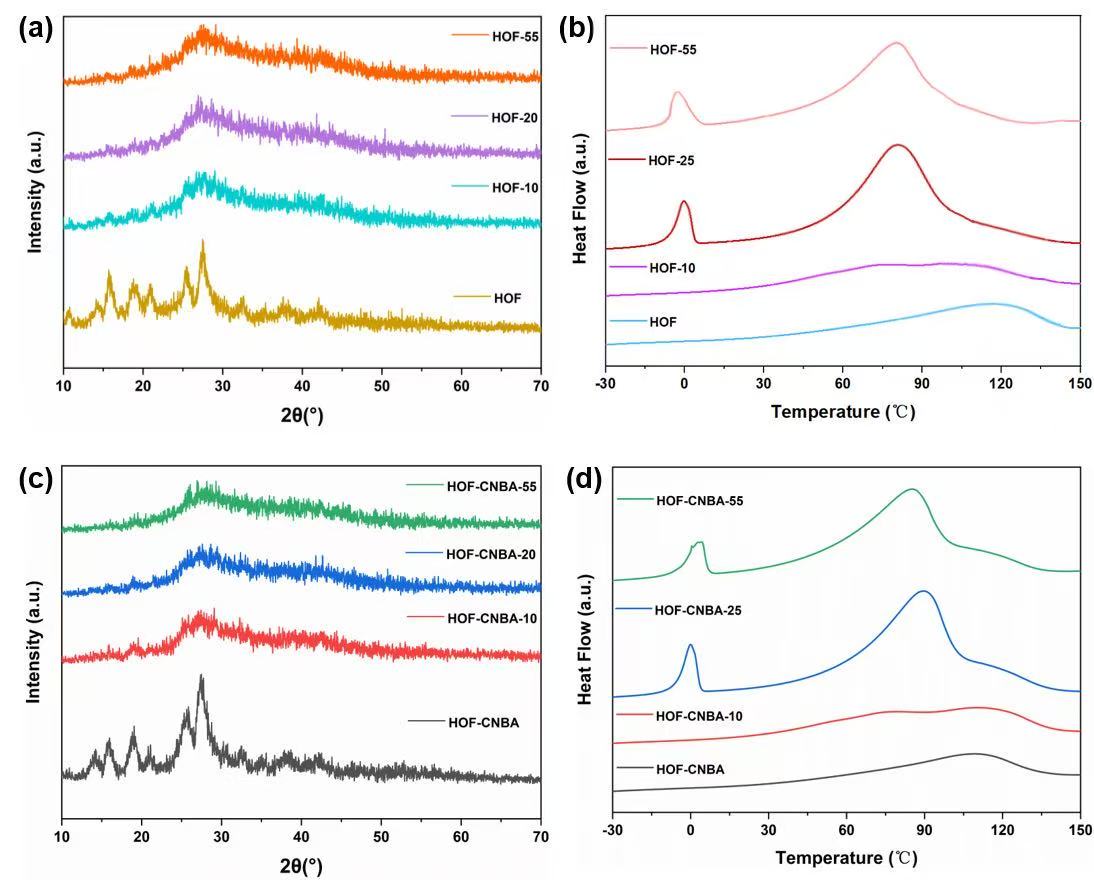


**Figure S16.** (a) XRD patterns of HOF with different water contents; (b) DSC curves of HOF with different water contents; (c) XRD patterns of HOF-CNBA with different water contents; (d) DSC curves of HOF-CNBA with different water contents.


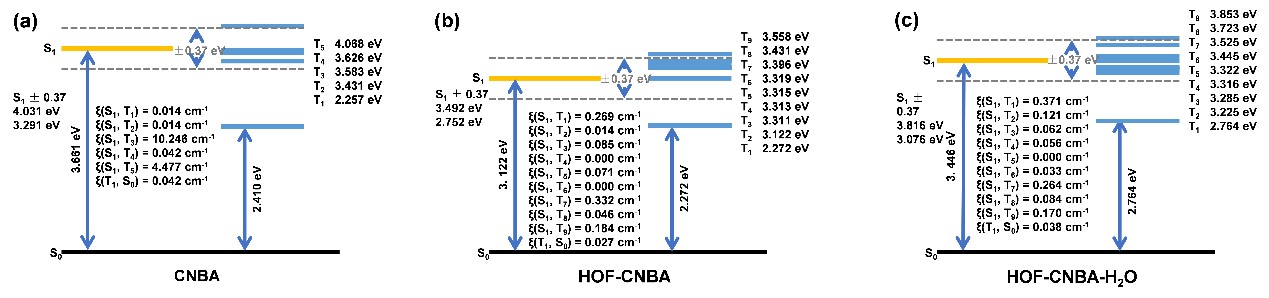


**Figure S17.** Vertical excitation energy levels (eV), possible ISC channels from S_1_ to T_n_ (indicated by lines), and SOC constants (cm^-1^) between singlet and triplet states for (a) CNBA, (b) HOF-CNBA, and (c) HOF-CNBA-H_2_O.


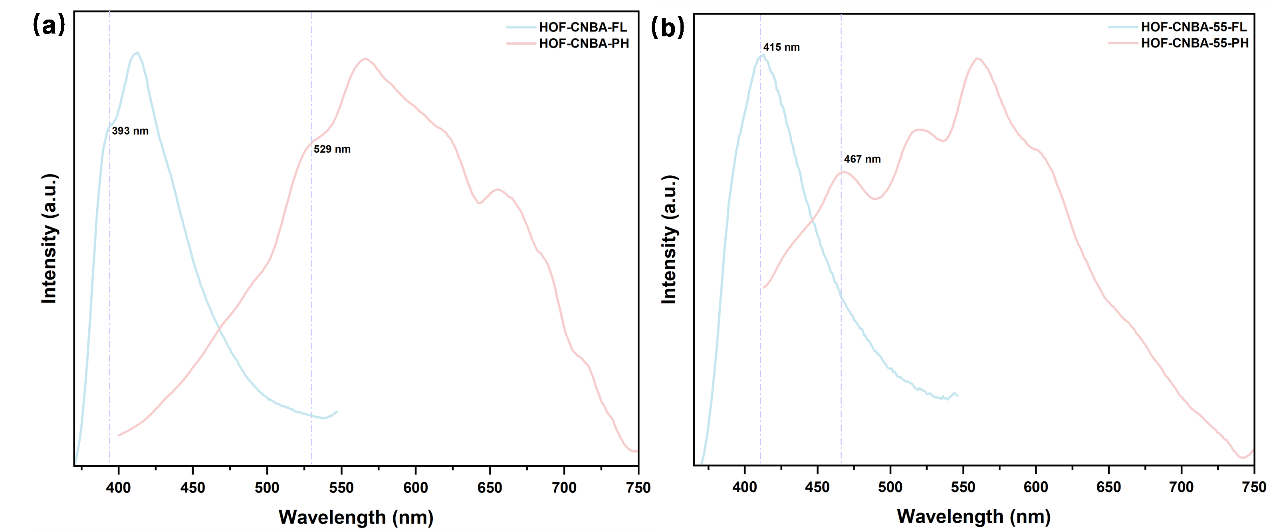


**Figure S18. Low-temperature (77 K) fluorescence (FL) and phosphorescence (PH) spectra of (a) HOF-CNBA and (b) HOF-CNBA-55.**


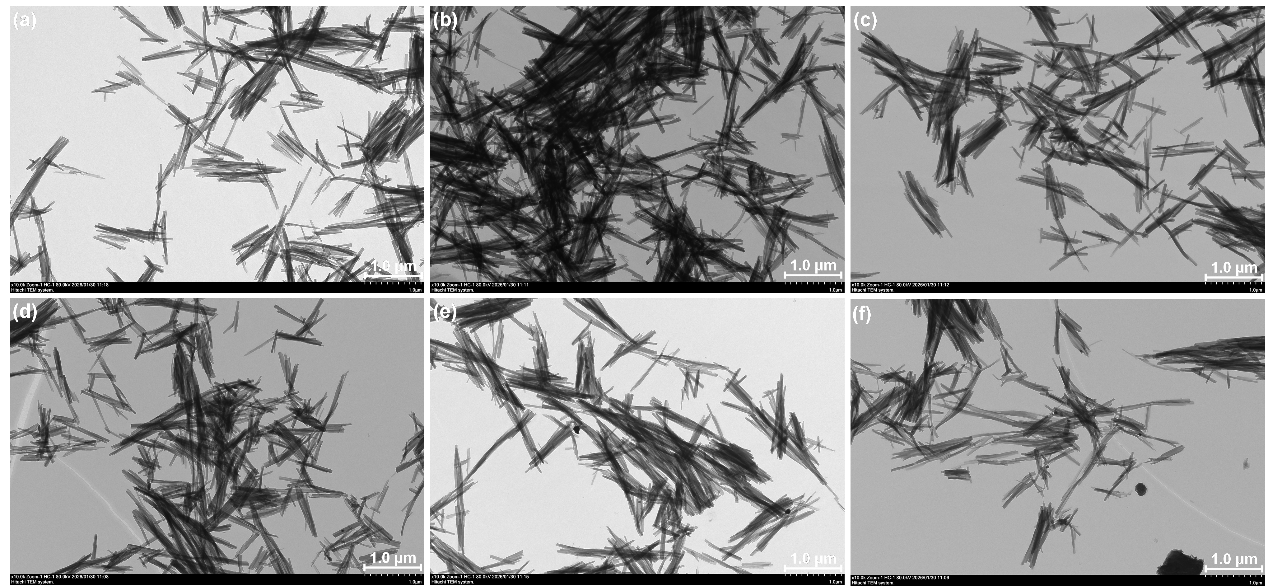


**Figure S19.** TEM images of (a) HOF, (b) HOF-PTH, (c) HOF-TCBA, (d) HOF-CNBA, (e) HOF-PBD, and (f) HOF-NTBD.


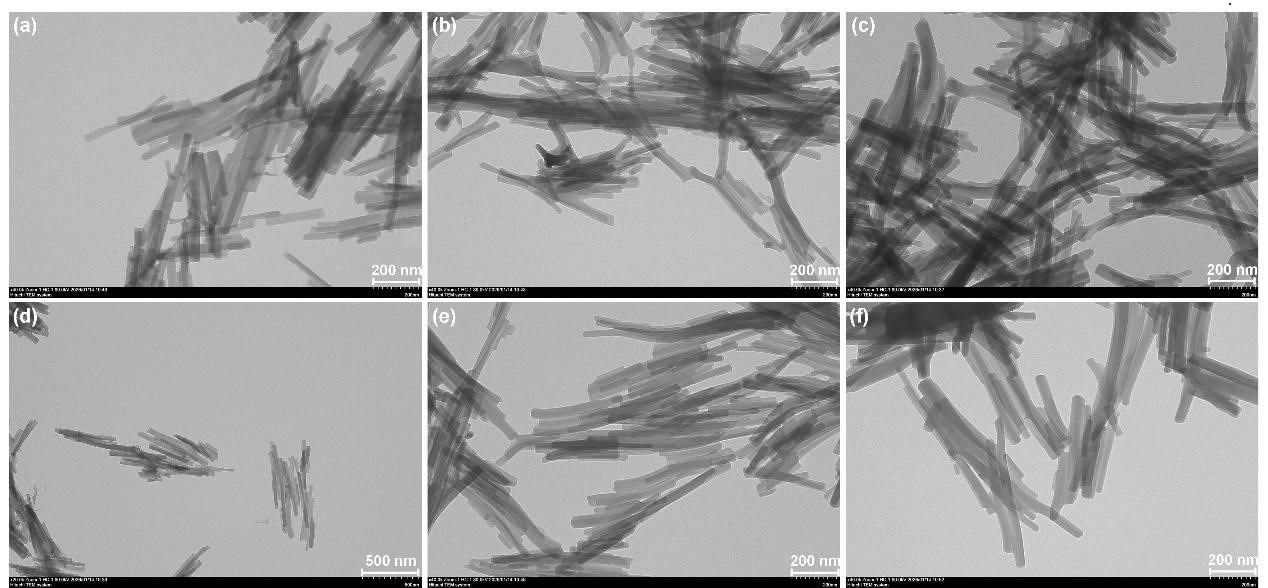


**Figure S20.** TEM images of (a) HOF, (b) HOF-PTH, (c) HOF-TCBA, (d) HOF-CNBA, (e) HOF-PBD, and (f) HOF-NTBD after regulating the reaction conditions.


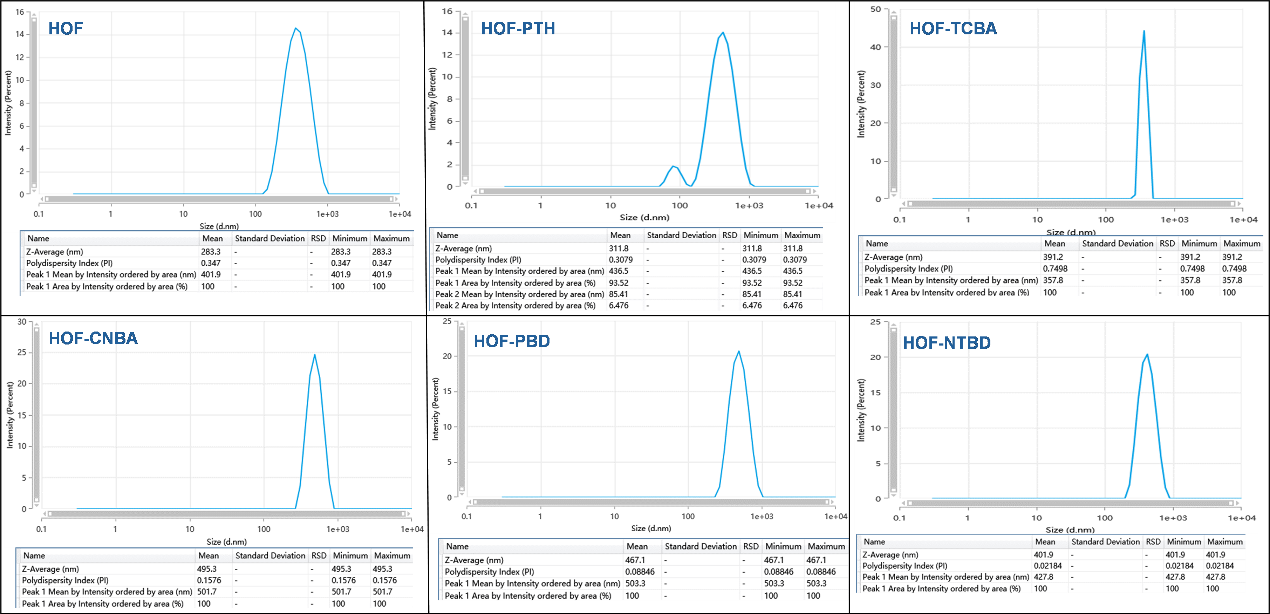


**Figure S21.** Dynamic light scattering (DLS) size distribution and key parameters of HOF materials. The intensity-weighted size distribution curves and corresponding parameters (Z-average diameter, polydispersity index (PDI), and peak size) are presented. All measurements were conducted in deionized water at a concentration of 0.1 mg/mL.


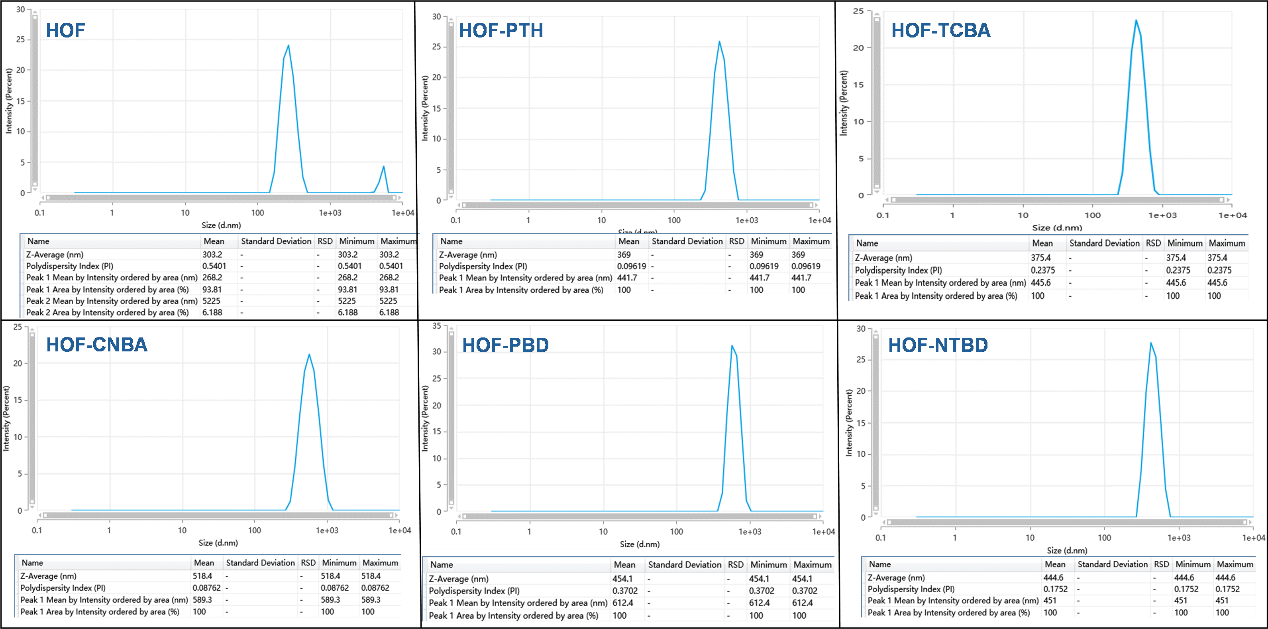


**Figure S22.** Dynamic light scattering (DLS) size distribution and key parameters of HOF materials after storage for 5 days. The intensity-weighted size distribution curves and corresponding parameters (Z-average diameter, polydispersity index (PDI), and peak size) are presented. All measurements were conducted in deionized water at a concentration of 0.1 mg/mL.


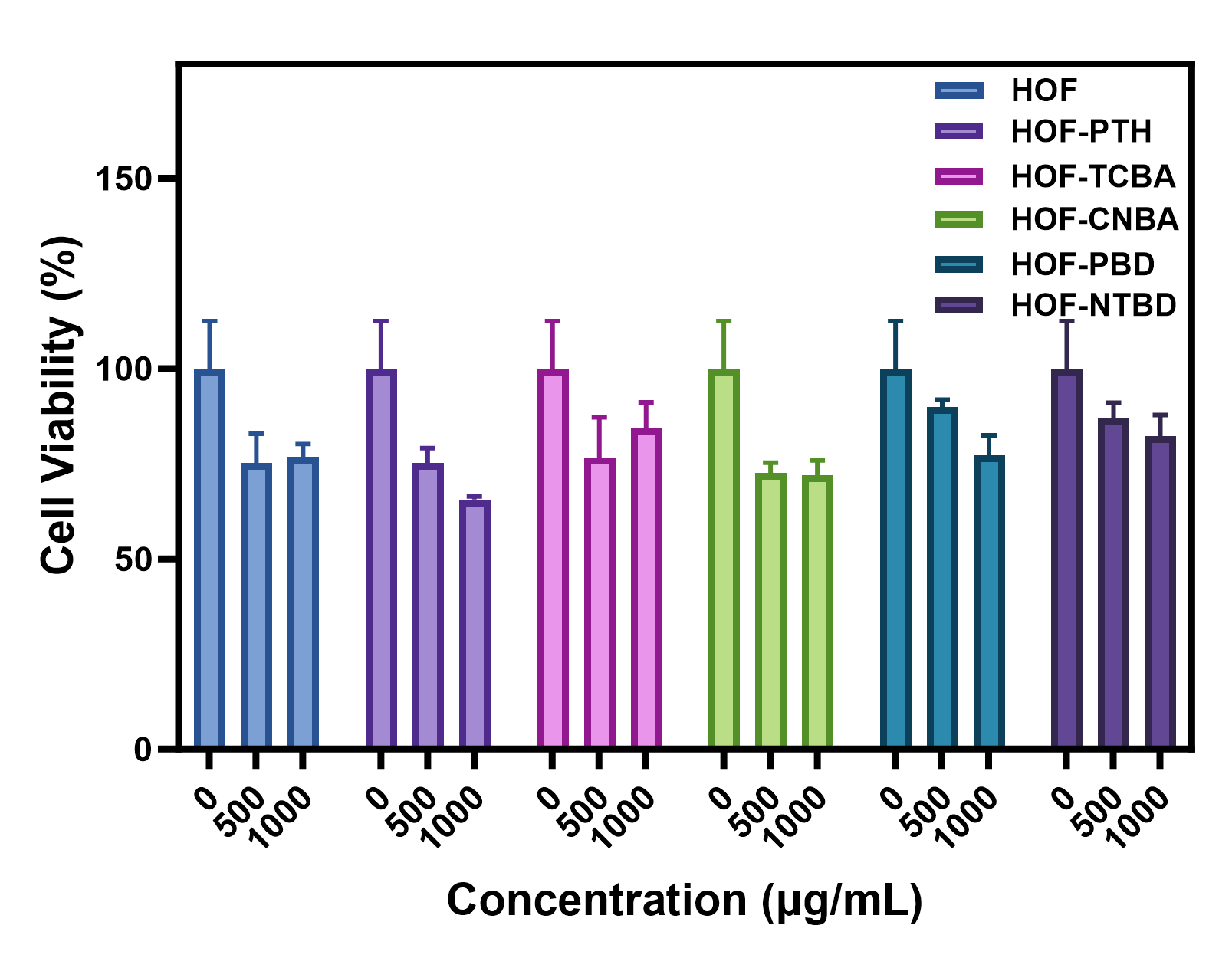


**Figure S23.** Cell viability of OCM-1 choroidal melanoma cells after incubation with materials at different concentrations (0, 500, 1000 μg/mL) for 24 h (CCK-8 assay).

**Table S1.** Photophysical Parameters of HOF-Guest Doped Materials.

| Sample | Fluorescence |  |  | Phosphorescence |  |  |
| --- | --- | --- | --- | --- | --- | --- |
|  | λ_em_ [nm] | Φ_F_ [%] | τ_F_ [ns] | λ_em_ [nm] | Φ_P_ [%] | τ_P_ [ms] |
| HOF | 334 | 24.8 | 311.5 | 508 | 3.4 | 42.73 |
| HOF-PTH | 400 | 18.5 | 480.3 | 500 | 11.8 | 209.82 |
| HOF-TCBA | 406 | 12.6 | 222.0 | 550 | 12.6 | 354.68 |
| HOF-CNBA | 392 | 9.7 | 206.8 | 568 | 9.7 | 83.76 |
| HOF-PBD | 400 | 11.4 | 204.5 | 531,620 | 11.4 | 132.47,78.18 |
| HOF-NTBD | 430 | 12.8 | 198.2 | 583,622 | 12.8 | 76.60,73.15 |

**Table S2.** Photophysical parameters of HOF-Guest doped materials after water doping.

| Sample | Fluorescence | Phosphorescence |  |
| --- | --- | --- | --- |
|  | Φ_F_ [%] | Φ_P_ [%] | τ_P_ [ms] |
| HOF-55 | 1.63 | 0.71 | 9.56 |
| HOF-CNBA-55 | 13.58 | 26.34 | 71.26 |

**References**

1. M. J. Frisch, G. W. Trucks, H. B. Schlegel, et al., “Gaussian 09,” 2009, Gaussian, Inc. Wallingford CT.
2. X. Gao, S. M. Bai, D. Fazzi, et al., “Evaluation of Spin-Orbit Couplings with Linear-Response Time-Dependent Density Functional Methods,” *Journal of Chemical Theory and Computation* 13 (2017): 515-524. <https://doi.org/10.1021/acs.jctc.6b00915>.
3. R. L. Martin, “Natural transition orbitals,” *The Journal of Chemical Physics* 118 (2003): 4775-4777, <https://doi.org/10.1063/1.1558471>.
